# A new genus and two new species of freshwater mussels (Unionidae) from western Indochina

**DOI:** 10.1038/s41598-019-39365-1

**Published:** 2019-03-11

**Authors:** Ekaterina S. Konopleva, John M. Pfeiffer, Ilya V. Vikhrev, Alexander V. Kondakov, Mikhail Yu. Gofarov, Olga V. Aksenova, Zau Lunn, Nyein Chan, Ivan N. Bolotov

**Affiliations:** 1 Northern Arctic Federal University, Arkhangelsk, Russia; 2 Federal Center for Integrated Arctic Research, Russian Academy of Sciences, Arkhangelsk, Russia; 3 Florida Museum of Natural History, University of Florida, Gainesville, USA; 4 Fauna & Flora International – Myanmar Program, Yangon, Myanmar

## Abstract

The systematics of Oriental freshwater mussels (Bivalvia: Unionidae) is poorly known. Here, we present an integrative revision of the genus *Trapezoideus* Simpson, 1900 to further understanding of freshwater mussel diversity in the region. We demonstrate that *Trapezoideus* as currently circumscribed is non-monophyletic, with its former species belonging to six other genera, one of which is new to science and described here. We recognize *Trapezoideus* as a monotypic genus, comprised of the type species, *T. foliaceus*. *Trapezoideus comptus*, *T. misellus*, *T. pallegoixi*, and *T. peninsularis* are transferred to the genus *Contradens*, *T. subclathratus* is moved to *Indonaia*, and *T. theca* is transferred to *Lamellidens*. *Trapezoideus prashadi* is found to be a junior synonym of *Arcidopsis footei*. *Trapezoideus dallianus*, *T. nesemanni*, *T. panhai*, *T. peguensis*, and two species new to science are placed in *Yaukthwa* **gen. nov**. This genus appears to be endemic of the Western Indochina Subregion. The two new species, *Yaukthwa paiensis* **sp. nov**. and *Y. inlenensis* **sp. nov**., are both endemic to the Salween River basin. Our results highlight that Southeast Asia is a species-rich freshwater mussel diversity hotspot with numerous local endemic species, which are in need of special conservation efforts.

## Introduction

Freshwater mussels (Unionoida) are a diverse and globally distributed clade^1,2^. There are two major freshwater mussel biodiversity hotspots, i.e. the Southeastern USA and East, South and Southeast Asia^3,4^. In comparison to the Southeastern USA, Asian freshwater mussel diversity is very poorly understood^3^. Several recent phylogenetic studies have substantially revised the taxonomy, morphological evolution, and historical biogeography of Asian freshwater mussels^4–13^ but many taxa remain poorly characterized. The genus *Trapezoideus* Simpson, 1900 has been included in several recent phylogenetic studies^5,8,12–14^ but taxon sampling within the genus remains largely incomplete, and no published study has yet to include the type species *Unio foliaceus* Gould, 1843^14^.

Konopleva *et al*.^14^ recently demonstrated that *Trapezoideus* was non-monophyletic with some of its representatives belonging to the subfamily Parreysiinae and other belonging to the Rectidentinae. Bolotov *et al*.^8^ revised the taxonomy of these two clades, describing the new genus *Trapezidens* (Parreysiinae: Lamellidentini) for the *Unio exolescens* group and suggested that *Trapezoideus* s. str. consisted of six species and was endemic to the rivers of western Indochina. However, that circumscription of *Trapezoideus* s. str. was based on morphological studies of the type species and an incomplete sample of other species previously attributed to *Trapezoideus*.

Recent collections of putative *Unio foliaceus* and several other species previously assigned to *Trapezoideus* (*Trapezoideus comptus*, *T. misellus*, *T. pallegoixi*, and *T. subclathratus*), as well as several morphologically similar specimens collected from the poorly characterized Salween River provide the basis for a more robust revision of the genus *Trapezoideus* and the tribe Contradentini more generally.

## Results

### Polyphyly of the genus *Trapezoideus*

**Figure 1:**
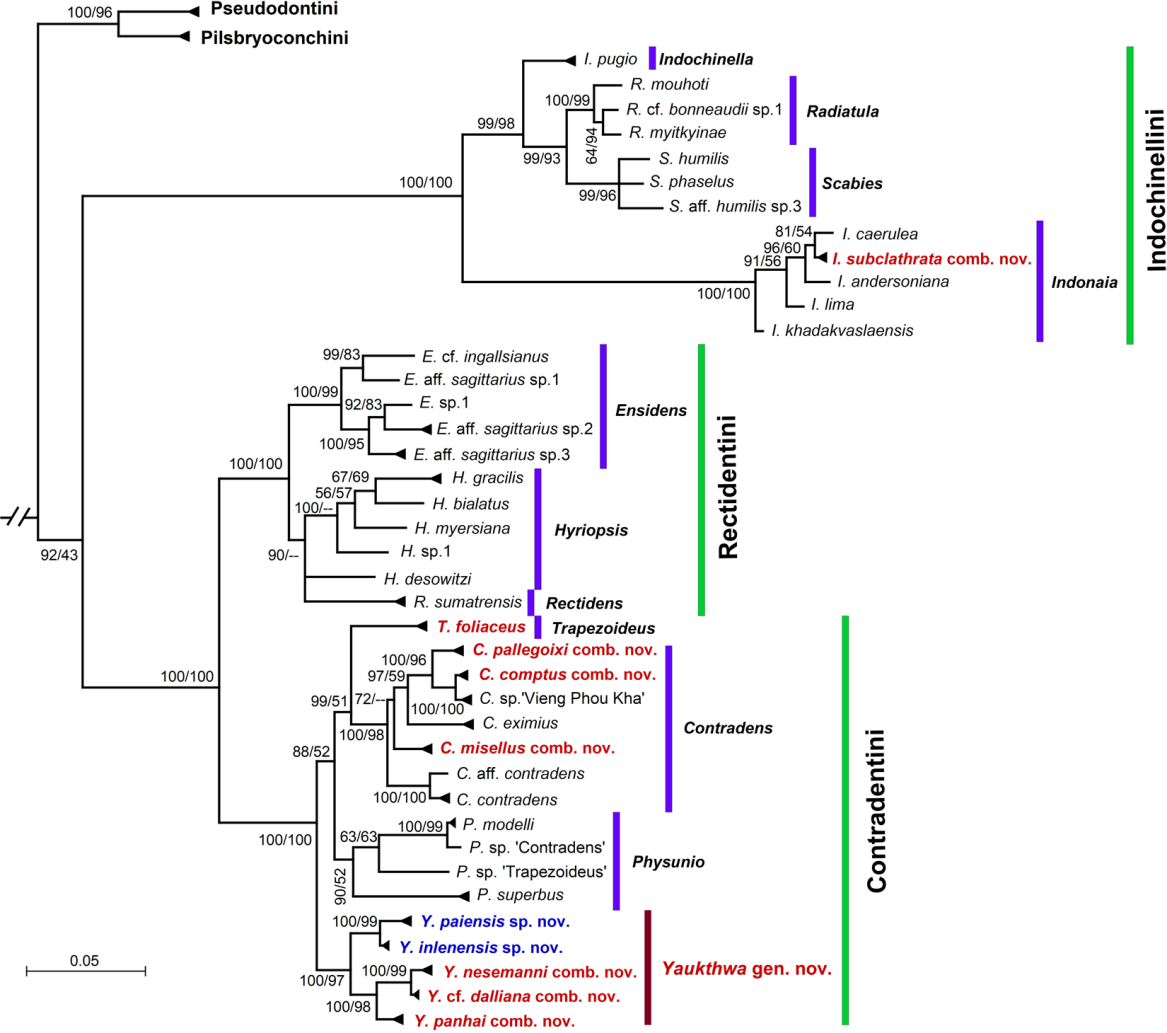
Fifty-percent majority rule consensus phylogenetic tree recovered from Bayesian inference analysis of the complete data set of mitochondrial and nuclear sequences of the Unionidae species (five partitions: three codons of *COI* + *16 S rRNA* + *28 S rRNA*). *Margaritifera dahurica* and *Gibbosula laosensis* were used as an outgroup (not shown). Scale bar indicates the branch lengths. Black numbers near nodes are Bayesian posterior probabilities/ML bootstrap support values. The taxa previously assigned to *Trapezoideus* are colored red to highlight their non-monophyly. The two species new to science are colored blue.

**Figure 2:**
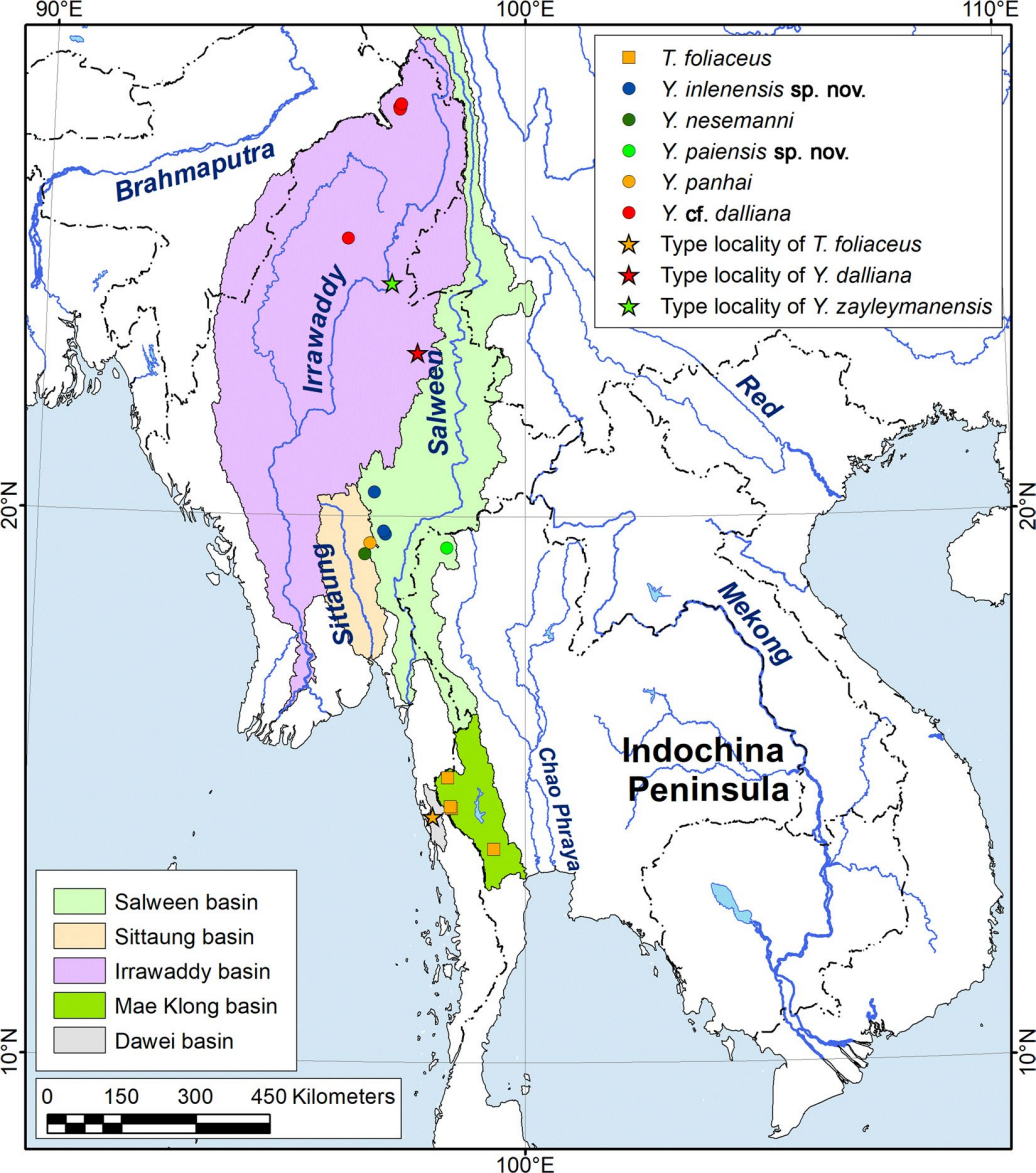
Distribution ranges of *Trapezoideus foliaceus* and species in the genus *Yaukthwa* **gen. nov**. The corresponding river basins are highlighted in color. The map was developed using ESRI ArcGIS 10 software (www.esri.com/arcgis). The topographic base of the map was compiled with Natural Earth Free Vector and Raster Map Data (www.naturalearthdata.com), GSHHG version 2.3.7 (http://www.soest.hawaii.edu/pwessel/gshhg)^47^, and the HydroSHEDS database (http://www.hydrosheds.org)^48,49^. (Map: Mikhail Yu. Gofarov).

Our multi-locus phylogeny based on the mitochondrial *cytochrome c oxidase subunit I* (*COI*), *small ribosomal RNA* (16* S rRNA*), and the nuclear *large ribosomal RNA* (28* S rRNA*) gene fragments clearly indicates that the genus *Trapezoideus* sensu Bolotov *et al*. 2017 in its current understanding is a polyphyletic entity (Fig. 1; Supplementary Table 1). *Trapezoideus foliaceus*, the type species of this genus, represents a separate phylogenetic lineage within the tribe Contradentini. Five species from western Indochina, i.e. *Trapezoideus panhai*, *T. nesemanni*, *T*. cf. *dallianus* [=*T. subclathratus* sensu Bolotov *et al*.^8^], and two undescribed species, form a well-supported and distinct genus-level clade, *Yaukthwa* **gen. nov**. (BPP/BS = 100/97), the range of which covers the Ayeyarwady, Sittaung and Salween river drainages (Fig. 2). Three other species from the Mekong Basin, i.e. *Trapezoideus misellus*, *T. comptus*, and *T pallegoixi*, cluster together with the members of the genus *Contradens*. Finally, the sequenced topotype specimens of *Trapezoideus subclathratus* belong to the Parreysiinae and this species was found to be a member of the genus *Indonaia*.

Additionally, we revised the taxonomic placement of *Trapezoideus peninsularis*, *T. theca* and *T. prashadi* by means of a morphological approach, because the molecular sequence data for these nominal taxa is still lacking. We suggest that *Trapezoideus peninsularis* is a member of the genus *Contradens*, *T. theca* belongs to *Lamellidens*, and *Trapezoideus prashadi* is a junior synonym of *Arcidopsis footei* (see Taxonomic Account for details). Biogeographic data also supports these conclusions (see Taxonomic Account and Discussion).

### Morphological analyses of *Trapezoideus foliaceus* and *Yaukthwa* gen. nov

**Figure 3:**
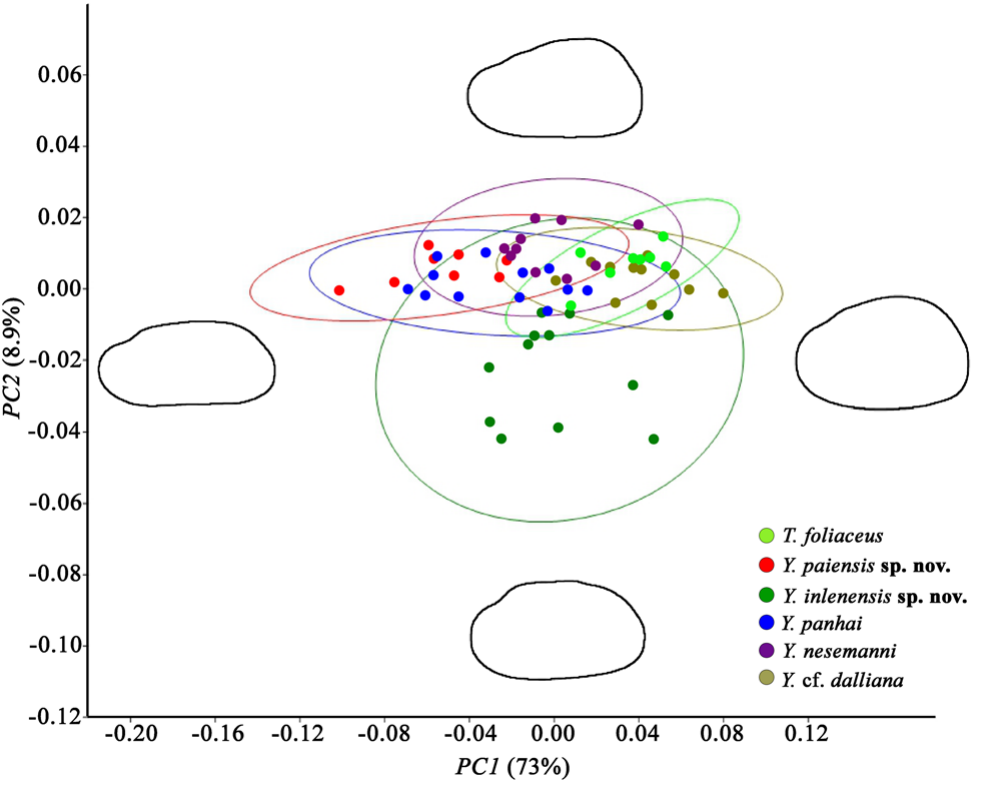
Scatter plot from principal component analysis (PCA) based on Fourier coefficients of the shell shape of *Trapezoideus foliaceus* and five *Yaukthwa* species. The color lines show 95% confidence ellipses. Colors correspond to biological species (see legend). The PC1 axis describes 73% and the PC2 axis describes 9% of a total variation. Synthetic outlines of “extreme” shell morphotypes are shown in four sides of the scatter plot.

**Figure 4:**
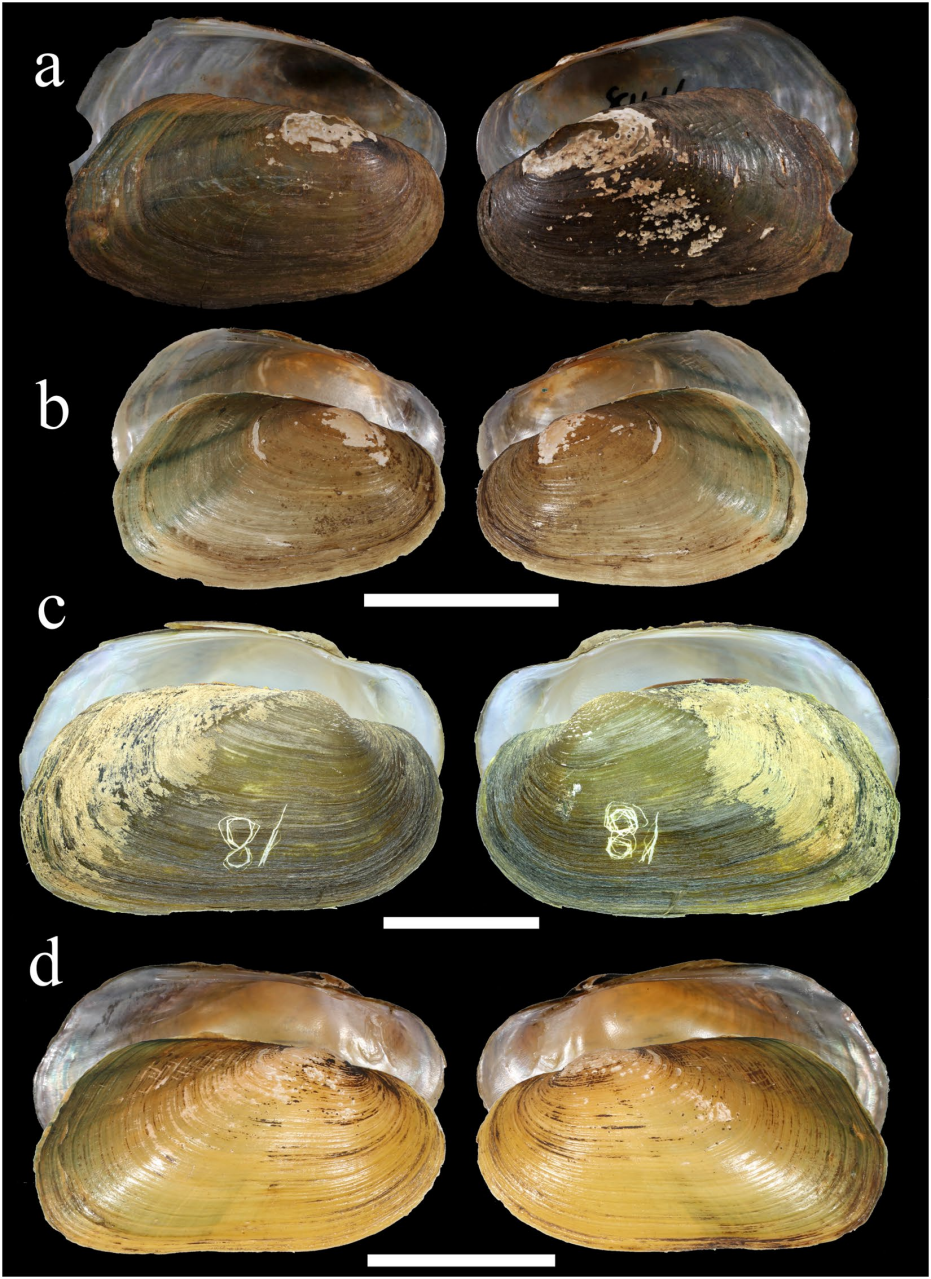
Shells of *Trapezoideus* and *Yaukthwa* species. (**a**) Lectotype of *Trapezoideus foliaceus*, Tavoy, British Burma (NMNH 84161). (**b**) *T. foliaceus*, Mae Klong River, western Thailand (UF 507865). (**c**) *Y. inlenensis* **sp. nov**., Salween River Basin, tributary of Nam Pilu River, Mway Stream, Myanmar (holotype RMBH biv139_18). (**d**) *Y. paiensis* **sp. nov**., Salween River Basin, Khong River, northwestern Thailand (holotype UF 505164). Scale bars = 2 cm. (Photos: NMNH, with permission of Dr. Ellen Strong [a], John M. Pfeiffer [b, d], and Ekaterina S. Konopleva [c]).

**Figure 5:**
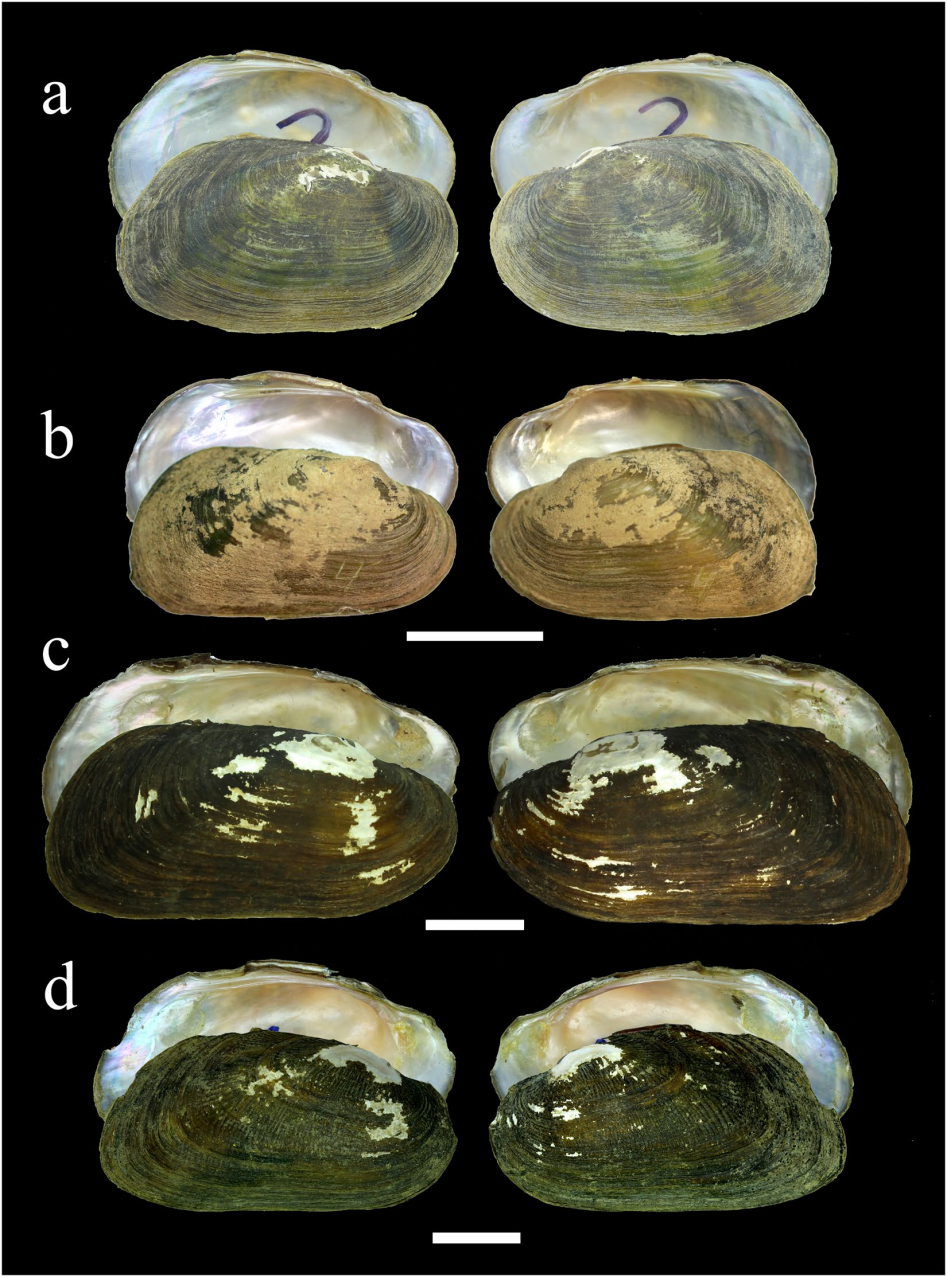
Shell morphology of *Yaukthwa* species and *Indonaia subclathrata* **comb. nov**. (**a**) *Yaukthwa* cf. *dalliana*, Nanuinhka Chaung River, Ayeyarwady Basin, Myanmar (RMBH biv111_2). (**b**) *Y. panhai* **comb. nov**., Kyan Hone River, Sittaung Basin, Myanmar (holotype NCSM 103033 [transferred from RMBH biv138_4^8^]). (**c**) *Y. nesemanni* **gen. & comb. nov**., Thauk Ye Kupt River, Sittaung Basin, Myanmar (holotype NCSM 103036 [transferred from RMBH biv255_2^8^]). (**d**) *Indonaia subclathrata* **comb. nov**., Chindwin River near Kalewa, Ayeyarwady Basin, Myanmar (topotype RMBH biv347_1). Scale bars = 2 cm [a-b, d] and 3 cm [c]. (Photos: Ekaterina S. Konopleva).

The primary diagnostic features of shell structure of the studied *Trapezoideus foliaceus* specimens from the Mae Klong River basin such as thin and trapezoidal shell, shallow anterior muscle attachment scars, and slender pseudocardinal teeth correspond well to the lectotype of this nominal taxon thought to be collected from the Dawei (Tavoy) River (Figs 2, 3, 4a,b). The other *Trapezoideus* representatives from western Indochina studied by us have more elongated shell (although small specimens are rather similar in shell shape), more developed hinge, well-marked anterior muscle scars even for young individuals, and more elevated umbo compared with those of *T. foliaceus* (Figs 4 and 5). These specimens have well distinguishable morphological features and belong to the *Yaukthwa* **gen. nov**.

Species of the *Yaukthwa* and *Trapezoideus* were also analyzed with respect to their shell contours (Fig. 3). For morphometric analysis, two significant principal components (PC1 and PC2) of the shell shape were obtained using a principal component analysis (PCA) approach based on 20 normalized Elliptic Fourier Descriptors (EFDs). PC1 axis describes 73% of the total variation of sagittal shell shape with much higher shell variability, while PC2 axis describes only 9%. The first component shows variation in the shell height, dorsal edge elevation and curve of ventral margin. The second component reflects the position and elevation of umbo and the shape of posterior end. Four synthetic outlines of the ‘extreme’ shell forms are shown in Fig. 3. According to the PCA results, 95% confident ellipses of all the studied species mainly overlapped, with exception of *Yaukthwa inlenensis* **sp. nov**., the specimens of which form a largely separate cloud.

**Taxonomic Account**

Family Unionidae Rafinesque, 1820

Subfamily Rectidentinae Modell, 1942

Tribe Contradentini Modell, 1942

Type genus: *Contradens* Haas, 1911 (by original designation)

**Genus** ***Trapezoideus*** **Simpson 1900**

Type species: *Unio foliaceus* Gould, 1843 (by original designation)

Comments: This genus was thought to comprise several species inhabiting numerous freshwater drainages from India to East Asia^4,15,16^, but we consider it to be a monotypic genus, with a rather local distribution range in western Thailand.

***Trapezoideus foliaceus*** **(Gould, 1843)**

*Unio foliaceus* Gould (1843): 141^17^.

*Trapezoideus foliaceus* Simpson (1900): 858^18^.

*Trapezoideus foliaceus* Konopleva *et al*. (2017): 214^14^.

Figure 4a,b

Type: Lectotype NMNH 84161.

Type locality: Tavoy, British Burma [Dawei River, Myanmar (approx. 14.50139° N, 98.15583° E)]^17^.

Material examined: UF 507697: Thailand, Mae Klong River basin, Pracham Mai River, 14.65983° N, 98.53422° E, 28.i.2015, 26 specimens (sequenced individuals = 2012–0443, 2012-0445), Pfeiffer & Page leg. UF 507702: Thailand, Mae Klong River basin, Song Karia River, 15.22318° N, 98.44648° E, 29.i.2015, 29 specimens (sequenced individuals = 2012-0457, 2012-0457), Pfeiffer & Page leg. UF 507865: Thailand, Mae Klong River basin, tributary of Pracham Mai River, 14.69334° N, 98.50639° E, 06.i.2017, 1 specimen (sequenced individual = ICH-02059), Pfeiffer & Page leg. UF507879: Thailand, Mae Klong River basin, Pachee River, 13.918134° N, 99.38227° E, 13.i.2017, 2 specimens (sequenced individuals = ICH-02104, ICH-02105), Pfeiffer & Page leg.

Redescription: Shell shape trapezoidal, inequilateral, not inflated, thin and small. Maximum shell length to 48.7 mm, height to 31.6 mm, width to 16.6 mm (*N* = 80). Posterior end broader than anterior one, somewhat oblique; anterior margin rounded. Umbo slightly elevated, corrugated; sculpture double-looped. Periostracum smooth, yellow-brown with green ribs on the posterior margin; nacre yellow-whitish. Pseudocardinal teeth slender and lamellar, two on the right valve and one on the left valve. Lateral teeth thin, elongated, slightly curved, one on the right valve and two on the left valve. Umbo cavity not deep. Muscle attachment scars shallow or reduced, oval-shaped.

Distribution: Only the type series is reported from “Tavoy” and there is some question as to the accuracy of the reported type locality (see discussion). All other records are from the Mae Klong Basin, western Thailand (Fig. 2).

Habitat: Common in small to medium sized streams and rivers with moderate current. Often found in coarse rocky substrate.

**Genus** ***Contradens*** **Haas, 1911**

Type species: *Unio contradens* Lea, 1838 (by original designation)

Comments: This genus is distributed in the Mekong Basin, Chao Phraya River, rivers of the Malacca Peninsula and Greater Sunda Islands^8,10^. Records from other areas most likely represent misidentified specimens (Supplementary Table 2).

***Contradens peninsularis*** **(Simpson, 1900) comb. nov**.

*Trapezoideus peninsularis* Simpson (1900): 859^18^.

Type: Not traced.

Type locality: Sumatra^18^.

Distribution: Sumatra, Indonesia.

Comments: Molecular sequence data for this poorly known species is still lacking. We transfer it to the genus *Contradens* on the basis of available biogeographic information^7–9,13^ and morphological data, although this taxonomic hypothesis requires further research.

***Contradens comptus*** **(Deshayes & Jullien, 1874) comb. nov**.

*Unio comptus* Deshayes & Jullien (1874): 126, pl. 6, Figs 3–4 ^19^.

*Diplodon ludovicianum* Rochebrune (1881): 43^20^.

*Trapezoideus misellus* Haas (1969): 76^21^.

*Trapezoideus exolescens comptus* Brandt (1974): 300^15^.

*Harmandia munensis* Brandt (1974): 284^15^.

*Trapezoideus comptus* Pfeiffer *et al*. (2018): 3^13^.

Type: Syntype MNHN IM-2000-1661.

Type locality: Peam Chelang, Cambodge [Mekong River, Peam Chilang village, Tboung Khmum District, Kampong Cham Province, Cambodia (approx. 12.0937° N, 105.5331° E)]^19^.

Distribution: Mekong Basin in Laos, Thailand, and Cambodia.

Comments: Our molecular phylogeny (Fig. 1) indicates that the specimens morphologically identified as *T. comptus* belong to the genus *Contradens* and should be transferred from *Trapezoideus* to *Contradens*.

***Contradens pallegoixi*** **(Sowerby, 1867) comb. nov**.

*Anodon pallegoixi* Sowerby (1867): pl. 8, sp. 18, fig. 17^22^.

*Trapezoideus pallegoixi* Simpson (1900): 859^18^.

Type: Holotype BMNH 1965193.

Type locality: Siam^22^.

Distribution: Mun River drainage (Mekong Basin) in Thailand.

Comments: This species is transferred to *Contradens* based on the multi-locus molecular data (Fig. 1).

***Contradens misellus*** **(Morelet, 1865) comb. nov**.

*Unio misellus* Morelet (1865): 21^23^.

*Trapezoideus misellus* Simpson (1900): 859^18^.

Type: Holotype BMNH 93-2-4-1593.

Type locality: Siam^23^.

Distribution: Chao Phraya Basin in Thailand.

Comments: Our molecular phylogeny (Fig. 1) recovered this species as a *Contradens* member.

**Genus** ***Yaukthwa*** **gen. nov**.

Figures 2, 4c,d, 5a–c.

Type species: *Yaukthwa nesemanni* (Konopleva, Vikhrev & Bolotov, 2017) **gen. & comb. nov**.

Etymology: The name of this genus means “freshwater bivalve” (*yaukthwa*) in Burmese language.

Diagnosis: This genus represents a distinct phylogenetic clade, but is morphologically similar to the *Contradens* and *Trapezoideus*. However, adult representatives of *Yaukthwa* **gen. nov**. can be distinguished by wider and more rounded anterior end, straighter dorsal margin without developed wing, and shallow posterior muscle scar.

Description: Shell middle-sized, from obovate for juvenile specimens to trapezoidal for adults, inequilateral, rather compressed, of various thicknesses. Right valve with one lateral tooth and two linear pseudocardinal teeth. For some specimens teeth may be reduced, usually to one weak tubercle-like lateral tooth and one pseudocardinal tooth in each valve. Left valve with two somewhat curved lateral teeth and one pseudocardinal tooth. Anterior muscle scar well developed, oval-shaped. Posterior muscle scar shallow. Ectobranchous brooding in outer demibranches. Inner demibranch attached to visceral mass by its anterior end.

Distribution: Western Indochina (Ayeyarwady, Bago, Sittaung and Salween basins in Myanmar and Salween Basin in western Thailand).

**Figure 6:**
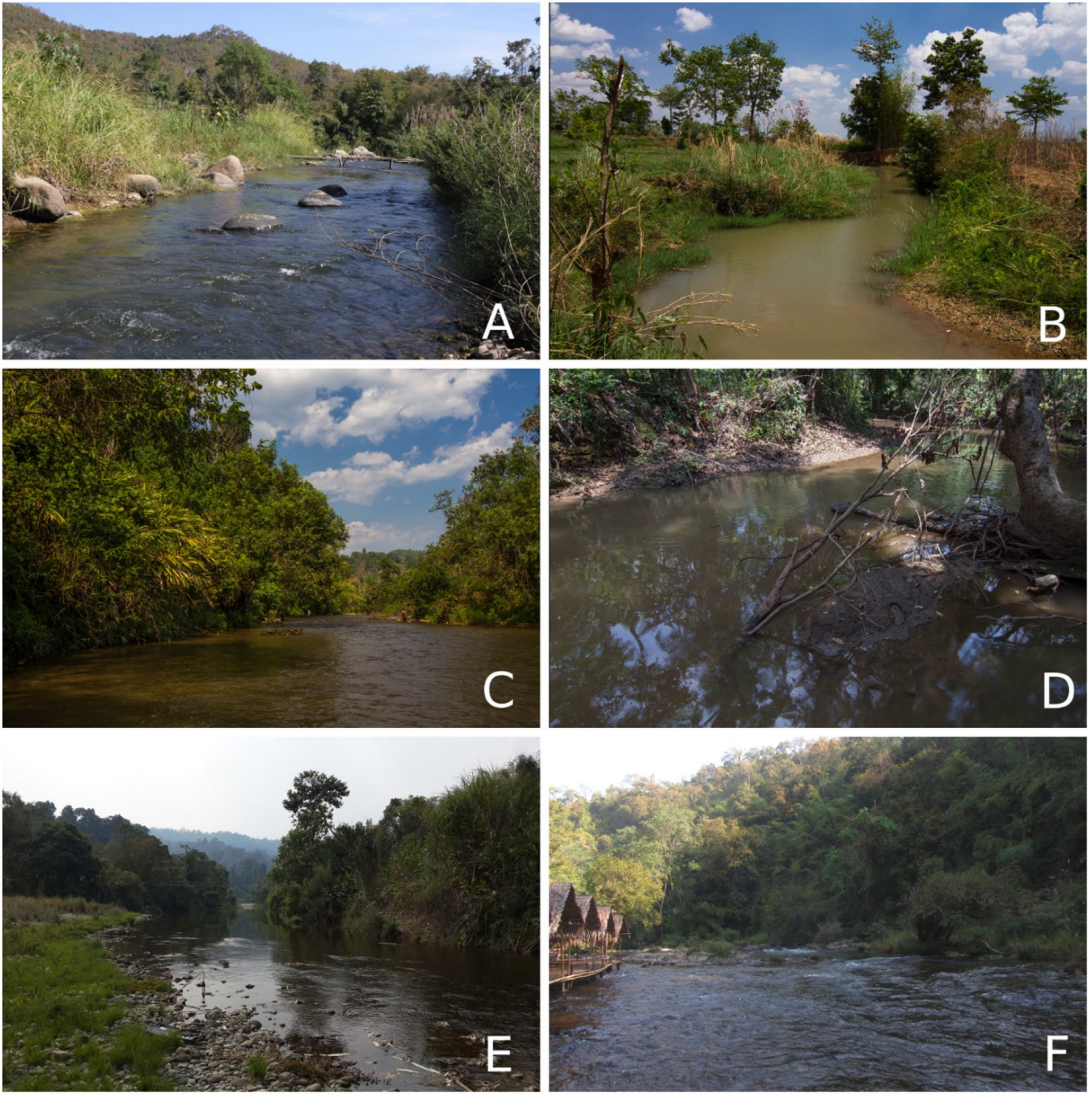
Type localities and habitats of the *Yaukthwa* species and habitat of *Trapezoideus foliaceus*. (**A**) Type locality of *Y. paiensis* **sp. nov**.: Khong River, Pai River basin, Salween Basin, northwestern Thailand. (**B**) Type locality of *Y. inlenensis* **sp. nov**.: Mway Stream, Salween Basin, Myanmar. (**C**) Habitat of *Y. nesemanni* **gen. & comb. nov**.: Thauk Ye Kupt River, Sittaung Basin, Myanmar. (**D**) Habitat of *Y. panhai* **comb. nov**.: Kyan Hone River, Sittaung Basin, Myanmar. (**E**) Habitat of *Y*. cf. *dalliana* **comb. nov**.: Nam Shu River, Malikha Basin, Ayeyarwady Drainage, Myanmar. (**F**) Habitat of *T. foliaceus*, Mae Klong Basin, western Thailand. (Photos: Zachary S. Randall [a, f] and Ilya V. Vikhrev [b-e]).

Habitat: Rapidly flowing mountain streams and rivers with sandy, gravel, and clay substrate, mostly within upland areas (Supplementary Table 3 and Fig. 6). However, *Yaukthwa inlenensis* **sp. nov**. can be found in rivers and streams with moderate current and clay substrate.

Comments: Here, we transfer five *Trapezoideus* taxa to the new genus and describe two additional species new to science.

***Yaukthwa nesemanni*** **(Konopleva, Vikhrev & Bolotov, 2017) gen. & comb. nov**.

*Trapezoideus nesemanni* Konopleva, Vikhrev & Bolotov (2017): 13, Fig. 5c^8^.

Type: Holotype NCSM 103033 [transferred from RMBH biv255_2^8^].

Type locality: Thauk Ye Kupt River, Sittaung Basin, Myanmar (19.3075° N, 96.7219° E)^8^.

Distribution: Known only from the type locality (Fig. 2).

***Yaukthwa panhai*** **(Konopleva, Bolotov & Kondakov, 2017) comb. nov**.

*Trapezoideus panhai* Konopleva, Bolotov & Kondakov (2017): 13, Fig. 5d^8^.

Type: Holotype NCSM 103036 [transferred from RMBH biv138_4^8^].

Type locality: Kyan Hone River, Sittaung Basin, Myanmar (19.5059° N, 96.8280° E)^8^.

Distribution: Known only from the type locality (Fig. 2).

***Yaukthwa dalliana*** **(Frierson, 1913) comb. nov**.

*Parreysia dalliana* Frierson (1913): 142^24^.

*Trapezoideus dallianus* Haas (1919): 263, pl. 32, Fig. 4 ^25,26^.

*Trapezoideus dallianus* Srinivasa Rao (1928): 464^27^.

?*Trapezoideus subclathratus* sensu Bolotov *et al*. (2017): 10^8^.

Type: Lectotype SMF 13699b (by present designation). Frierson^24^ stated that two specimens illustrated in Haas’s work [pl. 32, Figs 3 and 4]^25^ are representatives of his new species. Later, Haas^26^ revised the type series, and concluded that only the specimen in Fig. 4 should be the representative of *Trapezoideus dallianus*, and that the specimen on Fig. 3 belongs to *Trapezoideus foliaceus*. We agree that the latter specimen is conchologically different, and, at first glance, it may be a Lamellidentini member (e.g. *Trapezidens* sp.). It was collected from Pegu [Bago River, Myanmar]^26^. Haas^26^ also listed another specimen from the same lot (probably, specimen no. SMF 13699a) as an additional representative of *Trapezoideus dallianus*. However, this specimen was not pictured by Haas^25^, and was unknown to Frierson. Therefore, it could not be considered a part of the type series.

Type locality: Lashio-Fluss bei Lashio, nördliche Shan-Staaten [Lashio River near Lashio, Ayeyarwady Basin, northern Shan State, Myanmar (approx. 22.9946° N, 97.7650° E)]^26^.

Distribution: Known from the type locality (Fig. 2). Srinivasa Rao^27^ recorded two subfossil shells from the Namtu River at Hsenwi, 45 km NE of the type locality. Morphologically similar specimens were collected from the headwater of the Ayeyarwady River (Malikha Basin).

Comments: Our samples of *Trapezoideus subclathratus* sensu Bolotov *et al*.^8^ are morphologically similar to *Y. dalliana*, but they were collected from the Malikha River basin, far from the type locality of Frierson’s species (Fig. 2). However, the Malikha River belongs to the same drainage, the Ayeyarwady River. Here, we preliminary consider our samples as belonging to *Y*. cf. *dalliana*, but this hypothesis should be checked in a future based on molecular sequences of the topotypes from Lashio.

***Yaukthwa zayleymanensis*** **(Preston, 1912) comb. nov**.

*Trapezoideus foliaceus* var. *zayleymanensis* Preston (1912): 307^28^.

Type: Paratype SMF 3615.

Type locality: Bhamo [Bhamo, Ayeyarwady River (approx. 24.2669° N, 97.2210° E)]^28^.

Distribution: Known only from the type locality (Fig. 2) and from Zayleyman, Upper Burma^28,29^. We were unable to find an exact geographic position of Zayleyman, but the Upper Burma Region of British Empire included areas of the modern Shan and Kachin States of Myanmar. We suggest that Zayleyman was located somewhere on the Ayeyarwady River north of Mandalay.

Comments: Molecular data for this nominal taxon is still lacking. It is externally similar to *Yaukthwa nesemanni* **comb. nov**. from the Sittaung River by the elongated shell shape.

***Yaukthwa peguensis*** **(Anthony, 1865) comb. nov**.

*Unio peguensis* Anthony (1865): 351^30^.

Type: Holotype MCZ 161875.

Type locality: Pegu, British Burmah [Bago River, Myanmar]^30^.

Distribution: Known only from the type locality (Fig. 2).

Comments: Molecular data for this nominal taxon is still lacking.

***Yaukthwa paiensis*** **sp. nov**.

Figure 4d, Tables 1 and 2

**Table 1:** Shell measurements and reference DNA sequences for the type series of new *Yaukthwa* species from western Indochina.

Species	Status of Specimen	Specimen Voucher^*^	Shell Length, mm	Shell Height, mm	Shell Width, mm	NCBI’s GenBank acc. nos.		
						*COI*	*16* *S rRNA*	*28* *S rRNA*
*Y. inlenensis* **sp. nov**.	Holotype	RMBH biv_139_18	55.3	30.8	18.0	KX865924	KX865678	KX865795
	Paratype	RMBH biv_114_1	31.2	18.0	12.3	KX865915	KX865672	KX865786
	Paratype	RMBH biv_114_3	36.4	21.5	15.4	KX865916	KX865673	KX865787
	Paratype	RMBH biv_143_2	42.9	26.4	17.3	KX865917	KX865674	KX865788
	Paratype	RMBH biv_114_2	35.0	20.3	14.5	KX865918	KX865675	KX865789
	Paratype	RMBH biv_115_1	37.6	22.7	12.1	KX865919	n/a	KX865790
	Paratype	RMBH biv_115_3	33.4	20.2	14.2	KX865920	n/a	KX865791
	Paratype	RMBH biv_115_2	38.1	21.2	13.5	KX865921	n/a	KX865792
	Paratype	RMBH biv_139_7	40.7	23.0	13.8	KX865922	KX865676	KX865793
	Paratype	RMBH biv_139_15	50.2	29.3	17.2	KX865923	KX865677	KX865794
	Paratype	RMBH biv_140_22	46.0	26.2	19.1	KX865925	KX865679	KX865796
	Paratype	RMBH biv_140_24	43.7	24.4	18.9	KX865926	KX865680	KX865797
	Paratype	RMBH biv_140_25	46.5	25.6	19.1	KX865927	KX865681	KX865798
*Y. paiensis* **sp. nov**.	Holotype	UF 505164 (ICH_00638)	36.0	18.9	11.9	MH345970	MH346011	MH345991
	Paratype	UF 507709 (ICH_00639)	27.1	14.4	7.7	MH345971	MH346012	MH345992
	Paratype	UF 507709 (ICH_00640)	27.6	14.1	7.4	MH345972	n/a	n/a
	Paratype	UF 507709 (ICH_00637)	42.7	21.5	11.7	n/a	n/a	n/a
	Paratype	UF 507709	26.0	13.4	7.4	n/a	n/a	n/a
	Paratype	UF 507709	24.2	13.0	7.3	n/a	n/a	n/a
	Paratype	UF 507709	23.1	12.1	7.1	n/a	n/a	n/a
	Paratype	UF 507709	21.2	11.3	6.5	n/a	n/a	n/a

^*^RMBH – Russian Museum of Biodiversity Hotspots, Federal Center for Integrated Arctic Research, Russian Academy of Sciences, Arkhangelsk, Russia; UF – Florida Museum of Natural History, Gainesville, USA. n/a – not available.

**Table 2:** Molecular diagnoses of new *Yaukthwa* species from western Indochina.

Species	Mean COI *p*-distance from the nearest neighbor of new species, %	Nearest neighbor of new species	Fixed unique nucleotide differences based on the sequence alignment of congeners		
			*COI*	*16 S rRNA*	*28 S rRNA*
*Y. inlenensis* **sp. nov**.	3.3	*Y. paiensis* **sp. nov**.	77 G, 341 C, 344 C, 482 G	234 C, 249 A, 267 C, 464 G	None
*Y. paiensis* **sp. nov**.	3.3	*Y. inlenensis* **sp. nov**.	26 A, 296 A, 302 G, 317 A, 429 C, 461 T, 530 C, 579 C, 629 T	78 A, 239 T, 249 T, 254 C, 255 G, 319 C, 337 C, 448 T	121 C, 485 G, 761 C

Type material: Holotype UF 505164: Thailand, Salween River Drainage, Pai District, Mae Hong Son Province, Khong River, tributary of Pai River, off Rt. 1095, 19.4246° N, 98.4013° E, 10.i.2016, J. Pfeiffer & L. Page. Paratypes UF 507709: Thailand, Salween River Drainage, same locality and date as holotype, 7 specimens, J. Pfeiffer & L. Page.

Etymology: The species name is derived from the Pai River, the watershed from which the type specimen had been collected.

Diagnosis: *Yaukthwa paiensis* resembles its sister species, *Y. inlenensis*, but is distinguished by its more elongate shell outline, more parallel dorsal and ventral margins, less distinct umbo, and fixed nucleotide substitutions (Table 2).

Description: Shell outline subtrapezoidal, dorsal and ventral margins straight, dorsal margin elevated posteriorly, creating slight wing. Maximum length to 42.7 mm, height to 21.5 mm, width to 11.9 mm (Table 1). Posterior ridge broadly rounded, posterior slope gradual, often with very fine corrugations. Periostracum very smooth, yellow to brownish-yellow, often with green rays posteriorly. Nacre bluish-white, strongly iridescent posteriorly, often with orange tint near the umbo. Umbo only slightly elevated above hinge line. Pseudocardinal teeth strong, thin, elongate, one in each valve. Lateral teeth strong, thin, slightly curved, two in left valve, one in right. Umbo pocket, very shallow. Adductor muscle scars shallow, contiguous with pedal retractor scars.

Distribution: Known only from the type locality in northwestern Thailand (Fig. 2).

Habitat: The species inhabits slower flowing portions of the mountain stream, typically near sheltered and sandy banks. This species (and freshwater mussels in general) appears to be uncommon or patchily distributed in the Pai River system. No other freshwater mussel specimens (dead or alive) were found at any of our recent sampling sites in the Pai River watershed (*N* = 3) and there is only one other known freshwater mussel record from the Pai system (SMF 220825: 3 subfossil valves of *Gibbosula laosensis*)^15^.

***Yaukthwa inlenensis*** **sp. nov**.

*Trapezoideus* sp. ‘Salween’ sensu Bolotov *et al*. (2017): 10^8^

Figures 2 & 4c, Tables 1 and 2

Type material: Holotype RMBH biv139_18: Myanmar, Salween Basin, Mway Stream, a tributary of Nam Pilu River, 19.7266° N, 97.0992° E, 1.iv.2014, Vikhrev, Bolotov & locals leg. Paratypes: the type locality, 3 specimens (RMBH biv139_7, biv139_15, biv143_2), Myanmar, Salween Basin, Inle Lake channel, 20.4420° N, 96.9036° E, 1.iv.2014, 6 specimens (RMBH biv114_1, biv114_2, biv114_3, biv115_1, biv115_3, biv115_2), Myanmar, Salween Basin, Nam Pilu River, 3 specimens (RMBH biv140_22, biv140_24, biv140_25), 19.6746° N, 97.1352° E, 19.iv.2015, Bolotov & locals leg.

Etymology: The species name is derived from the Inle Lake, because it is widespread in tributaries and outlet of this water body.

Diagnosis: This species is very similar to *Yaukthwa paiensis*, but differs from it by more developed umbo and fixed nucleotide substitutions (Table 2).

Description: Shell shape variable, from elliptic to obovate, mainly with broader posterior side. Many specimens from the Nam Pilu River have constricted posterior end and more rounded ventral margin. All shells rather thick and inequilateral. Maximum shell length to 55.3 mm, height to 30.8 mm, width to 19.1 mm (Table 1). Umbo elevated with w-shaped sculpture, usually corrugated. Periostracum from olive-brown to dark-brown; the nacre whitish. Well-marked wrinkles grooves along the dorsal and posterior margin. Pseudo-cardinal teeth linear and strong, two on the right valve and one on the left valve. Lateral teeth thin, long and slightly curved, one on the right valve and two on the left valve. Umbo cavity rather deep. Anterior adductor scar oval-form and marked. Posterior adductor scar shallow or absent.

Distribution: Tributaries and the outlet of Inle Lake, Myanmar (Fig. 2).

Habitat: Moderately and slow flowing streams with clay and gravel substrate.

Subfamily Parreysiinae Henderson, 1935

Type genus: *Parreysia* Conrad, 1853

Tribe Lamellidentini Modell, 1942

Type genus: *Lamellidens* Simpson, 1900

**Genus** ***Lamellidens*** **Simpson, 1900**

Type species: *Unio marginalis* Lamarck, 1819 (by original designation)

***Lamellidens theca*** **(Benson, 1862) comb. nov**.

*Unio theca* Benson (1862): 186^31^.

*Trapezoideus theca* Simpson (1900): 859^18^.

Type: Not traced.

Type locality: Fluvio Cane, prope Banda, Bundelkhund [Ken River, near Banda, Uttar Pradesh, central India (approx. 25.4836° N, 80.3128° E)]^31^.

Distribution: Known only from the type locality.

Comments: Benson (p. 187)^31^ noted: “This shell, of which I found a single specimen, belongs to the *Corrianus* type of *Unio marginalis*, and is remarkable for its elongate-ovate non-rhomboidal form”. Benson’s protologue^31^ clearly indicates that this species is a member of the genus *Lamellidens*. The validity of this species is in doubt and deserves further research, because it is known from a single type specimen.

Tribe Indochinellini Bolotov, Pfeiffer, Vikhrev & Konopleva, 2018

Type genus: *Indochinella* Bolotov, Pfeiffer, Vikhrev & Konopleva, 2018 (by original designation)

**Genus** ***Indonaia*** **Prashad, 1918**

Type species: *Unio caeruleus* Lea, 1831 (by original designation)

***Indonaia subclathrata*** **(Martens, 1899) comb. nov**.

*Unio misellus* var. *subclathratus* Martens (1899): 44^32^.

*Trapezoideus misellus* var. *subclathratus* Subba Rao (1989): 195^16^.

Figure 5d

Type: Not traced.

Type locality: Im Chindwinfluss bei Kalewa und bei Matu < … > ; einige Stücke auch im Irawaddi selbst bei Yenangyoung [Chindwin River near Kalewa and Matu (approx. 23.1991° N, 94.3071° E), several specimens also from Ayeyarwady as far as Yenangyaung (approx. 20.4347° N, 94.8720° E)]^32^.

Distribution: Manipur and Chindwin rivers, and the middle reaches of the Ayeyarwady River, Myanmar.

Comments: This taxon has been considered a synonym of *Trapezoideus exolescens*^15,16^, but the latter species was found to be a member of the Lamellidentini^8,14^. Molecular analyses of the newly collected topotypes of *T. subclathratus* from Kalewa unexpectedly reveal that this species belongs to the genus *Indonaia*, representing another example of incorrect placement of the Parreysiinae taxa within the Rectidentinae based on an external resemblance of the shell^8,14^. Actually, *I. subclathrata* is externally quite similar to the *Yaukthwa* species (Fig. 5d).

Unionidae *incertae sedis*

**Genus** ***Arcidopsis*** **Simpson, 1900**

Type species: *Unio footei* Theobald, 1876 (by original designation)

***Arcidopsis footei*** **(Theobald, 1876)**

*Unio footei* Theobald (1876): 187, pl. 14, figs. 9-9a^33^.

*Trapezoideus prashadi* Haas (1922) **syn. nov**.: 101^34^.

Type: Holotype BMNH 88-12-4-1651 (*A. footei*); holotype SMF 3614 (*T. prashadi*).

Type locality: Kistna flumine prope ‘Gutparba falls’ [Gokak Falls, Ghataprabna River, Krishna Basin, southwestern India (approx. 16.1921° N, 74.7776° E)]^33^.

Distribution: Krishna River drainage in Western Ghats, India.

Comments: The type locality of *T. prashadi* is Mysore, Südostindien [Krishna Basin, Mysuru, Karnataka, southwestern India (approx. 12.4003° N, 76.6929° E)]^34^. Haas^25^ and Prashad^35^ listed the type specimen of *T. prashadi* as *A. footei*. Haas^34^ described this specimen as a new *Trapezoideus* species, but used only a brief description and figures of *A. footei*^33^ to delineate these taxa. Unfortunately, the figures in Theobald’s protologue [pl. 14, figs. 9-9a]^33^ are hardly resemble the shells of *A. footei*. Later, Haas (1969) noted that *A. footei* could actually be a member of the *Trapezoideus* and may be conspecific to *T. prashadi*. From a conchological point of view, these taxa are identical, and the *Arcidopsis* appears to be a valid genus, which is not related to *Trapezoideus*. We therefore considered *T. prashadi* as a junior subjective synonym of *A. footei*.

## Discussion

In this study, *Trapezoideus* was not recovered as monophyletic, and its putative species were distributed across the tribe Contradentini (five distinct lineages in three genera) and one of its former species (*T. subclathratus*) is placed within the distantly related tribe Indochinellini (Fig. 1). On the basis of morphological and biogeographic patterns we remove three other species from the genus *Trapezoideus*, i.e. *T. peninsularis*, *T. theca*, and *T. prashadi*. We briefly discuss each of these hypotheses in terms of their systematic and biogeographic relevance (see Taxonomic Account).

We collected putative specimens of the type species of *Trapezoideus*, *T. foliaceus*, from several headwater sites of the Mae Klong Basin in western Thailand, which is directly adjacent to the Dawei (Tavoy) Drainage, the drainage from which *Unio foliaceus* is presumed to be described from (Fig. 2). However, in the description of *Unio foliaceus* Gould^17^ never explicitly mentions the type locality of the species. The specimens are presumed to be from Tavoy on the basis that Rev. F. Mason, a missionary in the region, sent the specimens to Gould^14^. It is therefore possible that the specimens did not in fact originate from that river. The sequenced Mae Klong specimens are morphologically very similar to the lectotype of *Unio foliaceus* (Fig. 4a,b) and we consider them representatives of this nominal taxon. The inclusion of specimens of the type species of the genus *Trapezoideus* provided the material necessary to more completely revise the tribe Contradentini. Our *Trapezoideus foliaceus* specimens are recovered as the sister lineage to the genus *Contradens* (Fig. 1). The topology recovered here is completely consistent with the morphology-based hypothesis of Konopleva *et al*.^14^ who suggested that *Trapezoideus foliaceus* belongs to the Rectidentinae while *T. exolescens* belongs to the Parreyssiinae.

The taxa previously attributed to *Trapezoideus* from the Western Indochina Subregion belong to another genus, *Yaukthwa* **gen. nov**. This new genus comprises at least seven species, inhabiting the Salween, Sittaung, Bago and Ayeyarwady River drainages (Fig. 2). This new genus is morphologically similar to *Trapezoideus*, and their species are indistinguishable from each other by a morphometric shell shape analysis, with only the exception of *Yaukthwa inlenensis* **sp. nov**., showing a high variation in the shell shape, likely because of wider range of habitats. *Yaukthwa* **gen. nov**. is phylogenetically distant from the other Contradentini clades (*Trapezoideus*, *Physunio*, and *Contradens*) that are distributed east of the Salween – Mekong drainage divide, supporting a previously established biogeographic division of Southeast Asia^9^. In general, five freshwater mussel genera seem to be endemic to western Indochina: *Yaukthwa* **gen. nov**., *Indochinella*, *Pseudodon*, *Leoparreysia*, and *Trapezidens*.

All the *Yaukthwa* species and *Trapezoideus foliaceus* were recorded from lotic freshwater systems, i.e. rapidly flowing rivers and streams, mostly within upland areas (Supplementary Table 3). With respect to this evidence, dam construction, water pollution, and forest cutting appear to be the primary treats for their populations, as was shown for Laos, Borneo and Malaysia^10,11,36^. Taking into account local distribution ranges of the *Yaukthwa* and *Trapezoideus* species, they should be a focus of special conservation efforts from the governments, local authorities and local communities of Myanmar and Thailand, as well as international organizations. In conclusion, our results confirm a high conservation significance of the Oriental freshwater mussel fauna, because it includes numerous local endemic taxa. An integrative taxonomic approach becomes an essential tool for revisions of freshwater mussels in Southeast Asia.

## Methods

### Taxon sampling and molecular analysis

The samples of the Contradentini taxa were collected from Myanmar, Thailand, Laos, Cambodia, and Malaysia (Supplementary Table 1). Total genomic DNA extraction was carried out using NucleoSpin^®^ Tissue XS Kit (Macherey-Nagel GmbH & Co. KG, Germany), following the manufacturer’s protocol. For the molecular analyses, partial sequences of the *COI*, *16 S rRNA*, and *28 S rRNA* gene fragments were obtained and afterwards checked using a sequence alignment editor (BioEdit v. 7.2.5)^37^ as described in Bolotov *et al*.^8^ The PCR primers are provided in Supplementary Table 4.

### Phylogenetic analyses

Muscle algorithm implemented in MEGA6^38^ was used for sequence alignment of *COI*, *16 S rRNA* and *28 S rRNA* gene fragments. To get alignments with final lengths (Supplementary Table 5) we used GBlocks v. 0.91b^39^ as described in Bolotov *et al*.^8^ Through an online FASTA sequence toolbox (FaBox 1.41)^40^ we joined aligned data sets into combined nucleotide sequence alignments and collapsed them into unique haplotypes. Combined data set (3 codons of *COI* + *16 S rRNA* + *28 S rRNA*) of unique haplotypes was used for phylogenetic analyses. The best evolution models for each partition were selected based on the corrected Akaike Information Criterion (AICc) of MEGA6^38^ (Supplementary Table 6). Bayesian inference analysis (BI) was performed in MrBayes v. 3.2.6^41^ with four runs, each with three heated (temperature = 0.1) and one cold Markov chain, during 30 million generations and sampling every 1000th generation. The first 15% of trees were discarded as burn-in. All calculations were performed at San Diego Supercomputer Center through the CIPRES Science Gateway^42^. Trace analysis tool (Tracer v. 1.6)^43^ was used to check a convergence of the MCMC chains to a stationary distribution. The effective sample size (ESS) for each parameter was recorded as > 2000. The maximum likelihood (ML) analysis was performed in RAxML GUI v. 1.3 with 1000 bootstrap replications^44^. We used a unique GTR + G model for all the partitions.

### Morphological and morphometric analyses

We studied type series of nominal taxa and other shell lots in the collections of the BMNH – Natural History Museum, London, UK, NMNH – National Museum of Natural History, Smithsonian Institution, Washington, USA, MCZ – Museum of Comparative Zoology, Harvard University, Cambridge, USA, NCSM – North Carolina Museum of Natural Sciences, Raleigh, USA, UF – Florida Museum of Natural History, Gainesville, USA, SMF – Naturmuseum Senckenberg, Frankfurt, Germany, MNHN – Muséum national d’histoire naturelle, Paris, France, as well as RMBH – Russian Museum of Biodiversity Hotspots, Federal Center for Integrated Arctic Research, Russian Academy of Sciences, Arkhangelsk, Russia. The images of the nominal taxa from MUSSELp Database were also analyzed^45^. The comparative analysis of shell morphology was carried out with regard to the main distinguishing traits, such as shell shape, umbo position, structures of pseudo-cardinal and lateral teeth, as well as muscle attachment scars^8,14^. Three shell dimensions at each specimen of the studied taxa, i.e., the length, height, and width of the shell (all at the maximum diameter), were measured using calipers (±0.1 mm). Shell shape of *Trapezoideus foliaceus* and the *Yaukthwa* species were analyzed through Fourier coefficients using software package SHAPE v. 1.3^46^ as described in Konopleva *et al*.^14^. We used 139 individuals, from 8 to 12 shells per each species, depending on the number of available specimens. Photographs were obtained for mussels from our collections and were processed using GIMP v. 2.8.2 (www.gimp.org).

### Nomenclatural acts

The electronic edition of this article conforms to the requirements of the amended International Code of Zoological Nomenclature (ICZN), and hence the new names contained herein are available under that Code from the electronic edition of this article. This published work and the nomenclatural acts it contains have been registered in ZooBank (http://zoobank.org), the online registration system for the ICZN. The LSID for this publication is: urn:lsid:zoobank.org:pub:01AE2C5D-6857-4F76-B0FF-A15AAF76DDC8. The electronic edition of this paper was published in a journal with an ISSN, and has been archived and is available from PubMed Central.

## Supplementary information

Supplementary Info

## Data Availability

The type series of the new species are available in the malacological collections of the Russian Museum of Biodiversity Hotspots (RMBH), Federal Center for Integrated Arctic Research, Russian Academy of Sciences, Arkhangelsk, Russia (*Yaukthwa inlenensis* **sp. nov**.) and Florida Museum of Natural History (UF), Gainesville, USA (*Y. paiensis* **sp. nov**.). The sequences obtained in this study are available from the NCBI GenBank database. Species locality, accession numbers and vouchers for each specimen are presented in Supplementary Table 1 as well as Table 1.
