# Supplementary material for: A new genus and two new species of freshwater mussels (Unionidae) from western Indochina
Source: Sci Rep. 2019 Mar 11;9:4106. doi: 10.1038/s41598-019-39365-1 (PMC6411986; doi:10.1038/s41598-019-39365-1)
Supplement: Supplementary file 1 — Supplementary Info [file 41598_2019_39365_MOESM1_ESM.pdf]

SUPPLEMENTARY INFORMATION

A new genus and two new species of freshwater mussels (Unionidae) from western Indochina

Ekaterina S. Konopleva, John M. Pfeiffer, Ilya V. Vikhrev, Alexander V. Kondakov, Mikhail Yu. Gofarov, Olga V. Aksenova, Zau Lunn, Nyein Chan & Ivan N. Bolotov

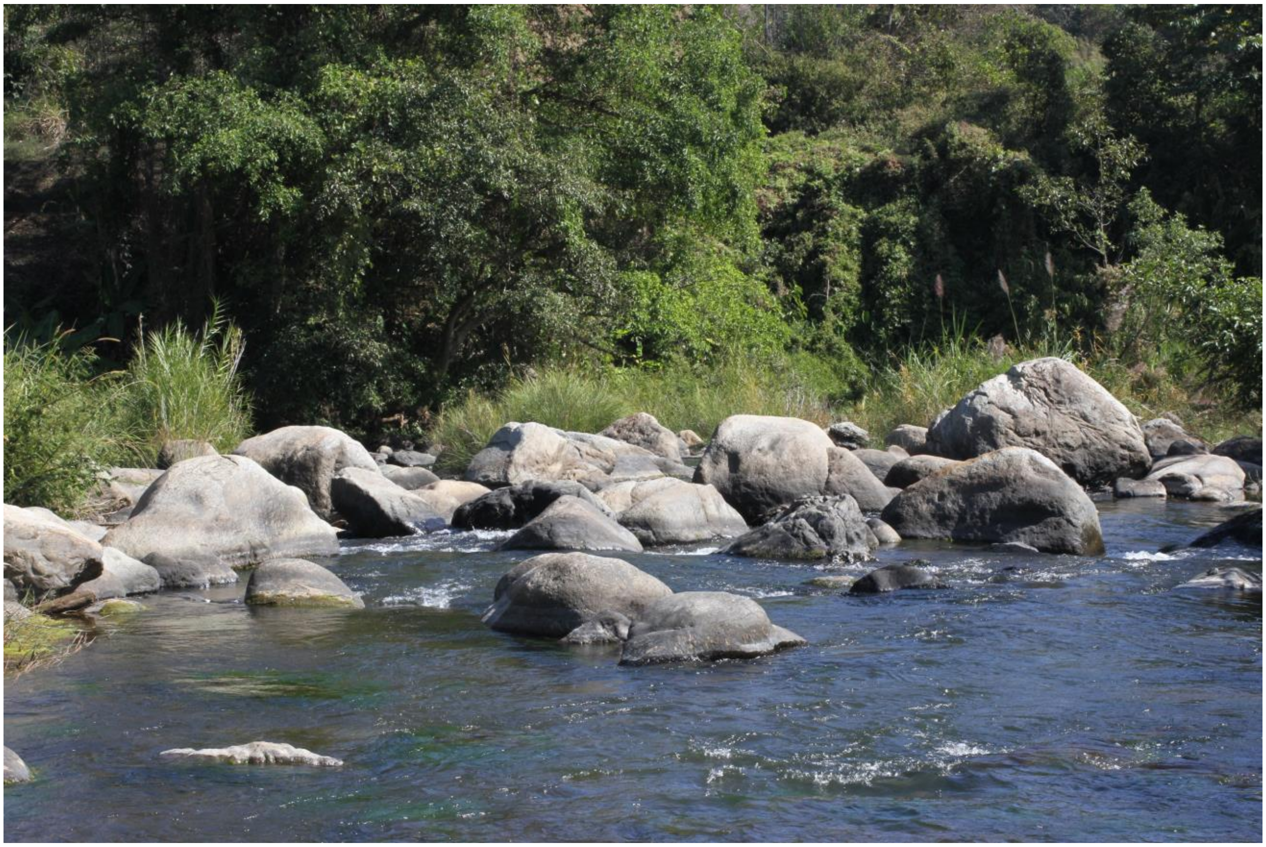

Habitat of *Yaukthwa paiensis* **sp. nov.**, Pai River basin, northwestern Thailand (Photo: Zachary S. Randall)

Contents

Supplementary Table 1. List of sequences used in this study, including the species, locality and NCBI's GenBank accession numbers

Supplementary Table 2. List of species in the tribe Contradentini Modell, 1942 (Unionidae: Rectidentinae)

Supplementary Table 3. Habitat characteristics for *Yaukthwa* spp.

Supplementary Table 4. Primer sequences for PCR amplification and sequencing

Supplementary Table 5. Alignment length prior to and after treatment for length variability in GBlocks v. 0.91b

Supplementary Table 6. Models of sequence evolution for each partition based on Akaike Information Criterion (AICc) of MEGA6

Supplementary References

Supplementary Table 1. List of sequences used in this study, including the species, locality and NCBI's GenBank accession numbers

Species	Locality	Specimen Voucher	*COI*	*16S rRNA*	*28S rRNA*
**PSEUDODONTINAE Frierson, 1927**					
**Pseudodontini Frierson, 1927**					
Pseudodon avae (Theobald, 1873)	Myanmar: Ayeyarwady River basin, a tributary of Lake Indawgyi	biv110_5	KX865858	KX865629	KX865730
P. avae (Theobald, 1873)	Myanmar: Ayeyarwady River basin, a tributary of Lake Indawgyi	biv110_10	KX865859	KX865630	KX865731
P. avae (Theobald, 1873)	Myanmar: Ayeyarwady River basin, a tributary of Lake Indawgyi	biv110_11	KX865860	KX865631	KX865732
P. bogani Bolotov, Kondakov & Konopleva, 2017	Myanmar: Sittaung River basin, Kanni River	biv 241/4	MF352216	MF352290	MF352348
P. bogani Bolotov, Kondakov & Konopleva, 2017	Myanmar: Sittaung River basin, Kanni River	biv 241/5	MF352217	MF352291	MF352349
P. bogani Bolotov, Kondakov & Konopleva, 2017	Myanmar: Sittaung River basin, Kanni River	biv 241/8	MF352218	MF352292	MF352350
P. manueli Konopleva, Kondakov & Vikhrev, 2017	Myanmar: Sittaung River basin, Pyowne River	biv 246/1	MF352228	MF352300	MF352358
P. manueli Konopleva, Kondakov & Vikhrev, 2017	Myanmar: Sittaung River basin, Pyowne River	biv 246/3	MF352229	MF352301	MF352359
P. manueli Konopleva, Kondakov & Vikhrev, 2017	Myanmar: Sittaung River basin, Pyowne River	biv 246/8	MF352230	MF352302	MF352360
**Pilsbryoconchini Bolotov, Vikhrev & Tumpeesuwan, 2017**					
Bineurus aff. *mouhotii* (Lea, 1863) sp.1	Laos: Mekong River basin, Nam Long River	biv_182/2	KX865876	KX865647	KX865747
B. aff. *mouhotii* (Lea, 1863) sp.2	Thailand: Mekong River basin, Loei River	biv_119/1	KX865879	KX865650	KX865750
B. aff. *mouhotii* (Lea, 1863) sp.3	Laos: Mekong River basin	UMMZ:304649	KP795026	KP795051	KP795009
Monodontina cambodjensis (Petit de la Saussaye, 1865)	Cambodia: Mekong River basin, Tonle Sap River: Pursat River	UMMZ:304350	KP795028	KF011262	KP795011
M. aff. *vondembuschiana* (Lea, 1840) sp.1	Thailand: Mekong River basin, Phong River	biv122	KX865861	KX865632	KX865733
M. aff. *vondembuschiana* (Lea, 1840) sp.2	Laos: Mekong River basin	UMMZ:304650	KP795029	KP795052	AF400694
Pilsbryoconcha compressa (Martens, 1860)	Thailand: Mekong River basin, artificial pond near the Ban Nong-Bua village	biv_116/1	KX865872	KX865643	KX865744
P. aff. *exilis* (Lea, 1838) sp.1	Cambodia: Mekong River basin	UMMZ:304647	KP795024	KP795049	KP795007
**PARREYSIINAE Henderson 1935**					
**Indochinellini Bolotov, Pfeifer, Vikhrev & Konopleva, 2018**					
Indochinella pugio (Benson, 1862)	Myanmar: Ayeyarwady River basin, Lake Nant Phar	biv_258/1	MF352261	MF352325	MF352386
I. pugio (Benson, 1862)	Myanmar: Ayeyarwady River basin, Lake Nant Phar	biv_258/2	MF352262	MF352326	MF352389
I. pugio (Benson, 1862)	Myanmar: Ayeyarwady River basin, Lake Myaung	biv_268/1	MF352285	n/a	n/a

Species	Locality	Specimen Voucher	*COI*	*16S rRNA*	*28S rRNA*
I. pugio (Benson, 1862)	Myanmar: Ayeyarwady River basin, Lake Myaung	biv_268/2	MF352286	MF352346	MF352403
I. pugio (Benson, 1862)	Myanmar: Ayeyarwady River basin, Lake Myaung	biv_268/4	MF352287	MF352347	MF352404
I. pugio (Benson, 1862)	Myanmar: Ayeyarwady River basin, Chindwin River: Paukin Lake	UA<USA-AL>:20739.1	JN243899	KP795046	JN243879
I. pugio (Benson, 1862)	Myanmar: Tavoy River	biv_147/3	KX865852	KX865623	KX865724
I. pugio (Benson, 1862)	Myanmar: Tavoy River	biv_147/10	KX865853	KX865624	KX865725
I. pugio (Benson, 1862)	Myanmar: Tavoy River	biv_147/18	KX865854	KX865625	KX865726
I. pugio (Benson, 1862)	Myanmar: Tavoy River	biv_148/4	KX865855	KX865626	KX865727
I. pugio (Benson, 1862)	Myanmar: Tavoy River	biv_148/7	KX865856	KX865627	KX865728
I. pugio (Benson, 1862)	Myanmar: Tavoy River	biv_148/15	KX865857	KX865628	KX865729
I. pugio (Benson, 1862)	Myanmar: Sittaung River basin, Myit Kyi Pauk stream	biv_ 251/1	MF352242	MF352312	MF352370
I. pugio (Benson, 1862)	Myanmar: Sittaung River basin, Myit Kyi Pauk stream	biv _251/2	MF352243	MF352313	MF352371
I. pugio (Benson, 1862)	Myanmar: Sittaung River basin, Myit Kyi Pauk stream	biv_251/3	MF352244	MF352314	MF352372
Indonaia caerulea (Lea, 1831)	India: Krishna River basin, fish pond	RRc1	KT869141	n/a	n/a
I. andersoniana (Nevill, 1877)	Myanmar: Ayeyarwady River basin, Lake Indawgyi	biv_108/1	KX865835	KX865606	KX865709
I. aff. *khadakvaslaensis* (Ray, 1966) sp.1	India	SBM7	KF690124	n/a	n/a
I. lima (Simpson, 1900)	India: Krishna River, Nagarjuna Sagar	RRl1	KP268827	n/a	n/a
I. subclathrata (Martens, 1899) **comb. nov.**	Myanmar: Manipur River	biv_338/1	MH700479	n/a	n/a
I. subclathrata (Martens, 1899) **comb. nov.**	Myanmar: Chindwin River	biv_347/1	MH700480	n/a	MH697870
Radiatula mouhoti Vikhrev, Bolotov & Konopleva, 2017	Myanmar: Sittaung River near Taungoo	biv_248/1	MF352234	MF352305	MF352363
R. cf. *bonneaudii* (Eydoux, 1838) sp.1	Myanmar: Ayeyarwady River basin, Tar Pein River	biv_260/5	MF352266	MF352330	MF352390
R. myitkyinae (Prashad, 1930)	Myanmar: Ayeyarwady River basin, Lake Indawgyi	biv_107/1	KX865838	KX865609	KX865710
Scabies humilis (Lea, 1856)	Thailand: Mekong River basin, Chi River	biv_126/1	KX865844	KX865615	KX865716
S. phaselus (Lea, 1856)	Thailand: Mekong River basin, Chi River	biv_124/1	KX865850	KX865621	KX865722
S. aff. *humilis* (Lea, 1856) sp.3	Cambodia: Mekong River basin	UMMZ:304646	KP795023	KP795048	KP795006
**RECTIDENTINAE Modell, 1942**					
**Contradentini Modell, 1942**					
Contradens contradens (Lea, 1838)	West Malaysia: Pahang River	ANSP:389059	DQ191411	n/a	AF400692
C. contradens (Lea, 1838)	Malaysia	X076	KX051294	n/a	n/a
C. contradens (Lea, 1838)	Malaysia	BIV1787	KX051270	n/a	n/a
C. contradens (Lea, 1838)	Malaysia	BIV1789	KX051269	n/a	n/a
C. contradens (Lea, 1838)	Malaysia	BIV1765	KX051265	n/a	n/a
C. contradens (Lea, 1838)	Malaysia	BIV1638	KX051259	n/a	n/a

Species	Locality	Specimen Voucher	*COI*	*16S rRNA*	*28S rRNA*
C. contradens (Lea, 1838)	Malaysia	X054	KX051251	n/a	n/a
C. contradens (Lea, 1838)	Malaysia	X060	KX051250	n/a	n/a
C. contradens (Lea, 1838)	Malaysia	X072	KX051247	n/a	n/a
C. contradens (Lea, 1838)	Malaysia	biv_211_2	MF352289	n/a	MF352406
C. contradens (Lea, 1838)	Malaysia	BIV1767	KX051253	n/a	n/a
C. contradens (Lea, 1838)	Malaysia	BIV1695	KX051249	n/a	n/a
C. contradens (Lea, 1838)	Malaysia	X132	KX051257	n/a	n/a
C. contradens (Lea, 1838)	Malaysia	X100	KX051246	n/a	n/a
C. contradens (Lea, 1838)	Malaysia	X208	KX051258	n/a	n/a
C. contradens (Lea, 1838)	Malaysia	BIV1764	KX051254	n/a	n/a
C. contradens (Lea, 1838)	Malaysia	BIV1777	KX051262	n/a	n/a
C. contradens (Lea, 1838)	Malaysia	BIV1748	KX051244	n/a	n/a
C. contradens (Lea, 1838)	Malaysia	BIV1709	KX051267	n/a	n/a
C. contradens (Lea, 1838)	Malaysia	BIV1757	KX051243	n/a	n/a
C. contradens (Lea, 1838)	Malaysia	BIV1710	KX051268	n/a	n/a
C. contradens (Lea, 1838)	Malaysia	BIV1839	KX051266	n/a	n/a
C. contradens (Lea, 1838)	Malaysia	BIV1639	KX051264	n/a	n/a
C. contradens (Lea, 1838)	Malaysia	X231	KX051261	n/a	n/a
C. contradens (Lea, 1838)	Malaysia	BIV1714	KX051256	n/a	n/a
C. contradens (Lea, 1838)	Malaysia	BIV1758	KX051255	n/a	n/a
C. contradens (Lea, 1838)	Malaysia	X43	KX051252	n/a	n/a
C. aff. *contradens* (Lea, 1838) sp.1	Cambodia: Mekong River basin	UMMZ:304652	KP795034	KP795054	KP795016
C. eximius (Lea, 1856)	Thailand: Mekong River basin, Chi River	biv_127 _1	KX865936	n/a	KX865807
C. eximius (Lea, 1856)	Thailand: Mekong River basin, Chi River	biv_127 _2	KX865937	n/a	KX865808
C. eximius (Lea, 1856)	Thailand: Mekong River basin, Chi River	biv_127 _3	KX865938	KX865686	KX865809
C. eximius (Lea, 1856)	Thailand: Mekong River basin, Phong River	biv_121 _1	KX865939	KX865687	KX865810
C. eximius (Lea, 1856)	Thailand: Mekong River basin, Phong River	biv_121_2	KX865940	KX865688	KX865811
C. eximius (Lea, 1856)	Thailand: Mekong River basin, Phong River	biv_121_3	KX865941	KX865689	KX865812
C. comptus (Deshayes & Jullien, 1874) **comb. nov.**	Thailand: Mekong River basin, Loei River	biv_119_5	KX865928	KX865682	KX865799
C. comptus (Deshayes & Jullien, 1874) **comb. nov.**	Thailand: Mekong River basin, Loei River	biv_119_6	KX865929	KX865683	KX865800
C. comptus (Deshayes & Jullien, 1874) **comb. nov.**	Laos: Mekong River basin, Nam Long River	biv_182_3	KX865930	KX865684	KX865801
C. comptus (Deshayes & Jullien, 1874) **comb. nov.**	Laos: Mekong River basin, Nam Long River	biv_182_12	KX865931	KX865685	KX865802
C. comptus (Deshayes & Jullien, 1874) **comb. nov.**	Laos: Mekong River basin, Nam Long River	biv_182_10	KX865932	n/a	KX865803
C. comptus (Deshayes & Jullien, 1874) **comb. nov.**	Laos: Mekong River basin, Nam Pe River	biv_184_1	KX865933	n/a	KX865804

Species	Locality	Specimen Voucher	*COI*	*16S rRNA*	*28S rRNA*
C. comptus (Deshayes & Jullien, 1874) **comb. nov.**	Laos: Mekong River basin, Nam Pe River	biv_184_3	KX865934	n/a	KX865805
C. comptus (Deshayes & Jullien, 1874) **comb. nov.**	Laos: Mekong River basin, Nam Pe River	biv_185_2	KX865935	n/a	KX865806
C. comptus (Deshayes & Jullien, 1874) **comb. nov.**	Cambodia: Mekong River basin, Tonle Kong River	ICH-00471	MH345973	MH346013	MH345993
C. comptus (Deshayes & Jullien, 1874) **comb. nov.**	Cambodia: Mekong River basin, Tonle Kong River	ICH-00474	MH345974	MH346014	MH345994
C. comptus (Deshayes & Jullien, 1874) **comb. nov.**	Cambodia: Mekong River basin, upstream of fishing village near the confluence of Tonle Sap River	ICH-00576	MH345975	MH346015	MH345995
C. comptus (Deshayes & Jullien, 1874) **comb. nov.**	Cambodia: Mekong River basin, upstream of fishing village near the confluence of Tonle Sap River	ICH-00577	MH345976	MH346016	MH345996
C. comptus (Deshayes & Jullien, 1874) **comb. nov.**	Thailand: Mekong River basin	ICH-00750	MH345977	MH346017	MH345997
C. comptus (Deshayes & Jullien, 1874) **comb. nov.**	Thailand: Mekong River basin	ICH-00752	MH345978	MH346018	MH345998
C. misellus (Morelet, 1865) **comb. nov.**	Thailand: Chao Phraya River basin, Ping River	2014-0685	MH345986	MH346026	MH346006
C. misellus (Morelet, 1865) **comb. nov.**	Thailand: Chao Phraya River basin, Ping River	2014-0687	MH345987	MH346027	MH346007
C. pallegoixi (Sowerby, 1867) **comb. nov.**	Thailand: Mekong River Basin, Mun River drainage, Pao river	2014-0613	MH345988	MH346028	MH346008
C. pallegoixi (Sowerby, 1867) **comb. nov.**	Thailand: Mekong River Basin, Mun River drainage, Pao river	2014-0614	MH345989	MH346029	MH346009
C. pallegoixi (Sowerby, 1867) **comb. nov.**	Thailand: Mekong River Basin, Mun River drainage, Pao river	2014-0616	MH345990	MH346030	MH346010
C. sp.'Vieng Phou Kha'	Laos: Mekong River basin, a tributary of Nam Fa River near Vieng Phou Kha	biv202_2	KY561630	KY561645	KY561662
C. sp.'Vieng Phou Kha'	Laos: Mekong River basin, a tributary of Nam Fa River near Vieng Phou Kha	biv203_5	KY561631	KY561646	KY561663
C. sp.'Vieng Phou Kha'	Laos: Mekong River basin, a tributary of Nam Fa River near Vieng Phou Kha	biv203_4	KY561632	KY561647	KY561664
Trapezoideus foliaceus (Gould, 1843)	Thailand: Mae Klong river basin, Pracham Mai River	2012-0443	MH345979	MH346019	MH345999
T. foliaceus (Gould, 1843)	Thailand: Mae Klong river basin, Pracham Mai River	2012-0445	MH345980	MH346020	MH346000
T. foliaceus (Gould, 1843)	Thailand: Mae Klong river basin, Song Karia River	2012-0457	MH345981	MH346021	MH346001
T. foliaceus (Gould, 1843)	Thailand: Mae Klong river basin, Song Karia River	2012-0458	MH345982	MH346022	MH346002
T. foliaceus (Gould, 1843)	Thailand: Mae Klong river basin, Tributary of Pracham Mai River	ICH-02059	MH345983	MH346023	MH346003
T. foliaceus (Gould, 1843)	Thailand: Mae Klong river basin, Pachee River	ICH-02104	MH345984	MH346024	MH346004
T. foliaceus (Gould, 1843)	Thailand: Mae Klong river basin, Pachee River	ICH-02105	MH345985	MH346025	MH346005
Physunio modelli Brandt, 1974	Thailand: Mekong River basin, Chi River	biv_125_2	KX865883	KX865654	KX865754
P. modelli Brandt, 1974	Thailand: Mekong River basin, Chi River	biv_125_3	KX865884	KX865655	KX865755

Species	Locality	Specimen Voucher	*COI*	*16S rRNA*	*28S rRNA*
P. modelli Brandt, 1974	Thailand: Mekong River basin, Chi River	biv_131_1	KX865885	n/a	KX865756
P. modelli Brandt, 1974	Thailand: Mekong River basin, Chi River	biv_131_2	KX865886	n/a	KX865757
P. modelli Brandt, 1974	Thailand: Mekong River basin, Chi River	biv_131_3	KX865887	n/a	KX865758
P. modelli Brandt, 1974	Thailand: Mekong River basin, Chi River	biv_131	KX865888	n/a	KX865759
P. modelli Brandt, 1974	Thailand: Mekong River basin, Phong River	biv205_1	KY561627	n/a	KY561659
P. modelli Brandt, 1974	Thailand: Mekong River basin, Phong River	biv205_2	KY561628	n/a	KY561660
P. modelli Brandt, 1974	Thailand: Mekong River basin, Phong River	biv205_3	KY561629	n/a	KY561661
P. sp.'Contradens'	Cambodia: Mekong River basin	UMMZ:304653	KP795035	KP795055	KP795017
P. sp.'Trapezoideus'	Laos: Mekong River basin, Nam Ou River	UMMZ:304347/UMMZ:MC:304347	KP795036	KF011265	KP795018
P. superbus (Lea, 1843)	Malaysia	X180	KX051282	n/a	n/a
P. superbus (Lea, 1843)	Malaysia	BIV1699	KX051280	n/a	n/a
P. superbus (Lea, 1843)	Malaysia	X236	KX051277	n/a	n/a
P. superbus (Lea, 1843)	Malaysia	X239	KX051276	n/a	n/a
P. superbus (Lea, 1843)	Malaysia	BIV1776	KX051278	n/a	n/a
P. superbus (Lea, 1843)	Malaysia	BIV1698	KX051281	n/a	n/a
P. superbus (Lea, 1843)	Malaysia	BIV1756	KX051279	n/a	n/a
P. superbus (Lea, 1843)	Malaysia	X246	KX051275	n/a	n/a
Yaukthwa nesemanni (Konopleva, Vikhrev & Bolotov, 2017) **gen. & comb. nov.**	Myanmar: Sittaung River basin, Thauk Ye Kupt River	biv_144_14	KX865906	KX865663	KX865777
Y. nesemanni (Konopleva, Vikhrev & Bolotov, 2017) **gen. & comb. nov.**	Myanmar: Sittaung River basin, Thauk Ye Kupt River	biv_144_25	KX865907	KX865664	KX865778
Y.a nesemanni (Konopleva, Vikhrev & Bolotov, 2017) **gen. & comb. nov.**	Myanmar: Sittaung River basin, Thauk Ye Kupt River	biv_144_19	KX865908	KX865665	KX865779
Y. nesemanni (Konopleva, Vikhrev & Bolotov, 2017) **gen. &comb. nov.**	Myanmar: Sittaung River basin, Thauk Ye Kupt River	biv_255_2	MF352254	n/a	MF352379
Y. nesemanni (Konopleva, Vikhrev & Bolotov, 2017) **gen. & comb. nov.**	Myanmar: Sittaung River basin, Thauk Ye Kupt River	biv_255_3	MF352255	n/a	MF352380
Y. nesemanni (Konopleva, Vikhrev & Bolotov, 2017) **gen. & comb. nov.**	Myanmar: Sittaung River basin, Thauk Ye Kupt River	biv_255_4	MF352256	n/a	MF352381
Y. panhai (Konopleva, Bolotov & Kondakov, 2017) **comb. nov.**	Myanmar: Sittaung River basin, Kyan Hone River	biv_138_4	KX865909	KX865666	KX865780
Y. panhai (Konopleva, Bolotov & Kondakov, 2017) **comb. nov.**	Myanmar: Sittaung River basin, Kyan Hone River	biv_138_7	KX865910	KX865667	KX865781
Y. panhai (Konopleva, Bolotov & Kondakov, 2017) **comb. nov.**	Myanmar: Sittaung River basin, Kyan Hone River	biv_155_4	KX865911	KX865668	KX865782
Y. panhai (Konopleva, Bolotov & Kondakov, 2017) **comb. nov.**	Myanmar: Sittaung River basin, Kyan Hone River	biv_155_25	KX865912	KX865669	KX865783

Species	Locality	Specimen Voucher	*COI*	*16S rRNA*	*28S rRNA*
Y. panhai (Konopleva, Bolotov & Kondakov, 2017) **comb. nov.**	Myanmar: Sittaung River basin, Kyan Hone River	biv_138_12	KX865913	KX865670	KX865784
Y. panhai (Konopleva, Bolotov & Kondakov, 2017) **comb. nov.**	Myanmar: Sittaung River basin, Kyan Hone River	biv_155_11	KX865914	KX865671	KX865785
Y. inlenensis **sp. nov.**	Myanmar: Salween River basin, Inle Lake Channel	biv_114_1	KX865915	KX865672	KX865786
Y. inlenensis **sp. nov.**	Myanmar: Salween River basin, Inle Lake Channel	biv_114_3	KX865916	KX865673	KX865787
Y. inlenensis **sp. nov.**	Myanmar: Salween River basin, Mway Stream	biv_143_2	KX865917	KX865674	KX865788
Y. inlenensis **sp. nov.**	Myanmar: Salween River basin, Inle Lake Channel	biv_114_2	KX865918	KX865675	KX865789
Y. inlenensis **sp. nov.**	Myanmar: Salween River basin, Inle Lake Channel	biv_115_1	KX865919	n/a	KX865790
Y. inlenensis **sp. nov.**	Myanmar: Salween River basin, Inle Lake Channel	biv_115_3	KX865920	n/a	KX865791
Y. inlenensis **sp. nov.**	Myanmar: Salween River basin, Inle Lake Channel	biv_115_2	KX865921	n/a	KX865792
Y. inlenensis **sp. nov.**	Myanmar: Salween River basin, Mway Stream	biv_139_7	KX865922	KX865676	KX865793
Y. inlenensis **sp. nov.**	Myanmar: Salween River basin, Mway Stream	biv_139_15	KX865923	KX865677	KX865794
Y. inlenensis **sp. nov.**	Myanmar: Salween River basin, Mway Stream	biv_139_18	KX865924	KX865678	KX865795
Y. inlenensis **sp. nov.**	Myanmar: Salween River basin, Nam Pilu River	biv_140_22	KX865925	KX865679	KX865796
Y. inlenensis **sp. nov.**	Myanmar: Salween River basin, Nam Pilu River	biv_140_24	KX865926	KX865680	KX865797
Y. inlenensis **sp. nov.**	Myanmar: Salween River basin, Nam Pilu River	biv_140_25	KX865927	KX865681	KX865798
Y. paiensis **sp. nov.**	Thailand: Salween River basin, Khong River	ICH-00638	MH345970	MH346011	MH345991
Y. paiensis **sp. nov.**	Thailand: Salween River basin, Khong River	ICH-00639	MH345971	MH346012	MH345992
Y. paiensis **sp. nov.**	Thailand: Salween River basin, Khong River	ICH-00640	MH345972	n/a	n/a
Y. cf. *dalliana* (Frierson, 1913)	Myanmar: Ayeyarwady River basin, Nanyinhka Chaung River	biv_111_2	KX865889	KX865656	KX865760
Y. cf. *dalliana* (Frierson, 1913)	Myanmar: Ayeyarwady River basin, Nanyinhka Chaung River	biv_111_21	KX865890	KX865657	KX865761
Y. cf. *dalliana* (Frierson, 1913)	Myanmar: Ayeyarwady River basin, Nanyinhka Chaung River	biv_111_43	KX865891	KX865658	KX865762
Y. cf. *dalliana* (Frierson, 1913)	Myanmar: Ayeyarwady River basin, Mali Hka River basin, Pan Khai stream	biv_101_4	KX865892	n/a	n/a
Y. cf. *dalliana* (Frierson, 1913)	Myanmar: Ayeyarwady River basin, Mali Hka River basin, Nam Shu River	biv_105_24	KX865893	n/a	n/a
Y. cf. *dalliana* (Frierson, 1913)	Myanmar: Ayeyarwady River basin, Mali Hka River basin, Pan Khai stream	biv_101_5	KX865894	KX865659	KX865763
Y. cf. *dalliana* (Frierson, 1913)	Myanmar: Ayeyarwady River basin, Mali Hka River basin, Pan Khai stream	biv_101_6	KX865895	n/a	KX865764
Y. cf. *dalliana* (Frierson, 1913)	Myanmar: Ayeyarwady River basin, Mali Hka River basin, Mansakun River	biv_103_17	KX865896	n/a	KX865765

Species	Locality	Specimen Voucher	*COI*	*16S rRNA*	*28S rRNA*
Y. cf. *dalliana* (Frierson, 1913)	Myanmar: Ayeyarwady River basin, Mali Hka River basin, Mansakun River	biv_103_18	KX865897	n/a	KX865766
Y. cf. *dalliana* (Frierson, 1913)	Myanmar: Ayeyarwady River basin, Mali Hka River basin, Mansakun River	biv_103_19	KX865898	n/a	KX865767
Y. cf. *dalliana* (Frierson, 1913)	Myanmar: Ayeyarwady River basin, Mali Hka River basin, Nam Balak River	biv_102_7	KX865899	KX865660	KX865768
Y. cf. *dalliana* (Frierson, 1913)	Myanmar: Ayeyarwady River basin, Mali Hka River basin, Nam Balak River	biv_102_11	KX865900	KX865661	KX865769
Y. cf. *dalliana* (Frierson, 1913)	Myanmar: Ayeyarwady River basin, Mali Hka River basin, Nam Balak River	biv_102_14	KX865901	KX865662	KX865770
Y. cf. *dalliana* (Frierson, 1913)	Myanmar: Ayeyarwady River basin, Mali Hka River basin, Nam Shu River	biv_105_31	KX865902	n/a	KX865771
Y. cf. *dalliana* (Frierson, 1913)	Myanmar: Ayeyarwady River basin, Mali Hka River basin, Nam Shu River	biv_105_32	KX865903	n/a	KX865772
Y. cf. *dalliana* (Frierson, 1913)	Myanmar: Ayeyarwady River basin, Mali Hka River basin, unnamed stream	biv_104_34	KX865904	n/a	KX865773
Y. cf. *dalliana* (Frierson, 1913)	Myanmar: Ayeyarwady River basin, Mali Hka River basin, unnamed stream	biv_104_35	KX865905	n/a	KX865774
Y. cf. *dalliana* (Frierson, 1913)	Myanmar: Ayeyarwady River basin, Mali Hka River basin, Pan Khai stream	biv_101_1	n/a	n/a	KX865775
Y. cf. *dalliana* (Frierson, 1913)	Myanmar: Ayeyarwady River basin, Mali Hka River basin, Nam Shu River	biv_105_22	n/a	n/a	KX865776
**Rectidentini Modell, 1942**					
Ensidens cf. *ingallsianus* (Lea, 1852)	Laos: Mekong River basin	NCSM84889	KX822641	n/a	KX822598
E. sp.1	Laos: Mekong River basin	NCSM84902	KX822642	n/a	KX822599
E. aff. *sagittarius* (Lea, 1856) sp.1	Cambodia: Mekong River basin	UMMZ:304651	KP795033	KP795053	KP795015
E. aff. *sagittarius* (Lea, 1856) sp.2	Thailand: Mekong River basin, artificial pond near the Ban Nong-Bua village	biv_117_1	KX865942	KX865690	KX865813
E. aff. *sagittarius* (Lea, 1856) sp.2	Thailand: Mekong River basin, artificial pond near the Ban Nong-Bua village	biv_117_2	KX865943	KX865691	KX865814
E. aff. *sagittarius* (Lea, 1856) sp.2	Thailand: Mekong River basin, artificial pond near the Ban Nong-Bua village	biv_117_3	KX865944	KX865692	KX865815
E. aff. *sagittarius* (Lea, 1856) sp.3	Thailand: Mekong River basin, Chi River	biv_128_1	KX865945	KX865693	KX865816
E. aff. *sagittarius* (Lea, 1856) sp.3	Thailand: Mekong River basin, Chi River	biv_128_2	KX865946	KX865694	KX865817
E. aff. *sagittarius* (Lea, 1856) sp.3	Thailand: Mekong River basin, Chi River	biv_128_3	KX865947	n/a	KX865818
E. aff. *sagittarius* (Lea, 1856) sp.3	Thailand: Mekong River basin, Chi River	biv_123_1	KX865948	n/a	KX865819
E. aff. *sagittarius* (Lea, 1856) sp.3	Thailand: Mekong River basin, Chi River	biv_123_2	KX865949	KX865695	KX865820
E. aff. *sagittarius* (Lea, 1856) sp.3	Thailand: Mekong River basin, Chi River	biv_123_3	KX865950	KX865696	KX865821
Hyriopsis gracilis Haas, 1910*	Thailand: Mekong River basin	n/a	KX822643	n/a	KX822600
H. gracilis Haas, 1910*	Thailand: Mekong River basin, Chi River	biv_130_1	KX865951	KX865697	KX865822
H. gracilis Haas, 1910*	Thailand: Mekong River basin, Chi River	biv_130_2	KX865952	KX865698	KX865823
H. gracilis Haas, 1910*	Thailand: Mekong River basin, Chi	biv_130_3	KX865953	n/a	KX865824

Species	Locality	Specimen Voucher	*COI*	*16S rRNA*	*28S rRNA*
	River				
H. sp.1	Thailand: Chao Phraya basin	839512HbB	KX383948	n/a	n/a
H. bialatus Simpson, 1900	Malaysia	BIV1775	KX051273	n/a	n/a
H. myersiana (Lea, 1856)	Thailand	n/a	KX822645	n/a	KX822602
H. desowitzi Brandt, 1974	Thailand	n/a	KX822644	n/a	KX822601
Rectidens sumatrensis (Dunker, 1852)	Malaysia	n/a	KX822664	n/a	KX822620
R. sumatrensis (Dunker, 1852)	Malaysia	biv 211_1	MF352288	n/a	MF352405
**Margaritiferidae (outgroup)**					
Gibbosula laosensis (Lea, 1863)	Laos: Mekong River basin, Nam Long River	biv 186_1	JX497731	KC845943	KT343741
Margaritifera dahurica (Middendorff,1850)	Far East of Russia: Amur River basin, Ilistaya River	biv 92_6	KJ161516	KJ943526	KT343747

*Do et al.[1] recommended using this name for what has been historically listed as *Hyriopsis bialatus* Simpson, 1900 only for the Mekong River basin populations.

Supplementary Table 2. List of species in the tribe Contradentini Modell, 1942 (Unionidae: Rectidentinae)

Genus	Species	Type locality	Distribution range
Trapezoideus Simpson 1900	*T. foliaceus* (Gould, 1843) [type species]	Tavoy, British Burmah	Mae Klong Basin, Thailand. The type series is reported from "Tavoy" (see Taxonomic Account)
Yaukthwa **gen. nov.**	*Y. nesemanni* (Konopleva, Vikhrev & Bolotov, 2017) **gen. & comb. nov.** [type species]	Thauk Ye Kupt River, Sittaung Basin, Myanmar	Thauk Ye Kupt River, Sittaung Basin, Myanmar
	**Y. dalliana* (Frierson, 1913) **comb. nov.**	Lashio River near Lashio, Ayeyarwady Basin, northern Shan State, Myanmar	Ayeyarwady Basin, Myanmar
	Y. inlenensis **sp. nov.**	Myanmar, Salween River drainage, Mway Stream, a tributary of Nam Pilu River	Lake Inle Region, Salween Basin, Myanmar
	Y. paiensis **sp. nov.**	Thailand, Salween River Drainage, Pai District, Mae Hong Son Province, Khong River, tributary of Pai River	Pai River, Salween Basin, Thailand
	Y. panhai (Konopleva, Bolotov & Kondakov, 2017) **comb. nov.**	Kyan Hone River, Sittaung Basin, Myanmar	Kyan Hone River, Sittaung Basin, Myanmar
	**Y. peguensis* (Anthony, 1865) **comb. nov.**	Pegu	Bago River, Myanmar
	Y. zayleymanensis (Preston, 1912) **comb. nov.**	Bhamo	Ayeyarwady Basin, Myanmar
Contradens Haas, 1911	*C. contradens* (Lea, 1838) [type species]	Java	Malaysia and probably Greater Sunda Islands
	C. comptus (Deshayes & Jullien, 1874) **comb. nov.**	Peam Chelang, Cambodia	Mekong Basin, Laos, Thailand and Cambodia
	C. eximius (Lea, 1856)	Siam	Mekong Basin, Thailand
	C. misellus (Morelet, 1865) **comb. nov.**	Siam	Chao Phraya Basin, Thailand
	C. pallegoixi (Sowerby, 1867) **comb. nov.**	Siam	Mun River, Mekong Basin, Thailand
	**C. fulvaster* (Drouet & Chaper, 1892)	Borneo	Borneo

Genus	Species	Type locality	Distribution range
	**C. inaequalis* (Rochebrune, 1881)	Sombor Rapids, Cambodia	Mekong Basin, Cambodia
	**C. peninsularis* (Simpson, 1900) **comb. nov.**	Sumatra	Sumatra, Indonesia
	**C. semmelinki* (Martens, 1891)	Tanah Laut, South Borneo	Borneo
	**C. gratiosus* (Philippi, 1843) [*Unio anceps* Deshayes in Deshayes & Jullien, 1874 was removed from the synonymy of *C. gratiosus* to the genus *Scabies* by Pfeiffer et al.[2]]	"New Holland" [erroneous]	Unknown
	**C. subcircularis* (Brandt, 1974) [most likely not a *Contradens* member, but a representative of the Leoparreysiini[2]]	Mekong River between Takek and Nakon Panom in Laos	Mekong Basin, Laos
	***C.* aff. *contradens* (Lea, 1838)	n/a	Mekong Basin, Cambodia
	***C.* "Vieng Phou Kha"	n/a	Mekong Basin, Laos
Physunio Simpson, 1900	*P. superbus* (Lea, 1843) [type species]	"New Holland" [erroneous; most probable type locality: the coast of Malacca, in the river Souzi]	Malaysia
	P. modelli Brandt, 1974	Thailand: Maenam Pong at Pong Nip Dam; Province Khon Kaen	Mekong Basin, Thailand
	**P. cambodiensis* (Lea, 1856)	Tackrong River, at Korat, Cambodia	Mekong Basin, Cambodia
	**P. inornatus* (Lea, 1856)	Siam	Mekong Basin, Thailand
	**P. micropterus* (Morelet, 1866)	Cambodia	Mekong Basin, Cambodia
	**P. friersoni* Simpson, 1914 [most likely a *Lamellidens* member]	Assam, India	Assam, India
	***P.* sp. "Contradens"	n/a	Mekong Basin, Cambodia
	***P.* sp. "Trapezoideus"	n/a	Mekong Basin, Laos

"*" indicates nominal taxa with an uncertain status that are in need of a further integrative revision; and "**" indicates species-level phylogenetic lineages that are still not attributed to a certain Linnean name. This table is compiled using the MUSSELp database[3]. n/a – not available.

Supplementary Table 3. Habitat characteristics for *Yaukthwa* spp.

Species	Habitat	Flow in microhabitat	Substrate
Y. paiensis **sp. nov.**	Mountain stream with rapids and runs	Slow	Sand, clay
Y. inlenensis **sp. nov.**	Mountain streams	Slow, moderate	Clay, gravel
Y. nesemanni **gen. & comb. nov.**	Mountain river	Moderately strong	Sand, gravel
Y. panhai **comb. nov.**	Mountain river	Moderately strong	Sand, gravel
Y. cf. *dalliana*	Mountain rivers and streams	Moderately strong	Sand, gravel

Supplementary Table 4. Primer sequences for PCR amplification and sequencing

Gene fragment	Primer's name	Direction	Sequence (5′-3′)	Reference
COI	LoboF1	Forward	kbtchacaaaycayaargayathgg	Ref. 4
	LoboR1	Reverse	taaacytcwggrtgwccraaraayca	
16S rRNA	16Sar	Forward	cgcctgtttatcaaaaacat	Ref. 5
	16sar-L-myt	Forward	cgactgtttaacaaaaacat	Ref. 6
	16sbr-H-myt	Reverse	ccgttctgaactcagctcatgt	
28S rRNA	C1	Forward	acccgctgaatttaagcat	Ref. 7
	D2	Reverse	tccgtgtttcaagacgg	

Supplementary Table 5. Alignment length prior to and after treatment for length variability in GBlocks v. 0.91b[8]

Partition	Original length of alignment (bp)	Fraction selected by GBlocks (%)	Final length of alignment (bp)
COI	660	100	660
28S rRNA	845	92	775
16S rRNA	513	87	445

Supplementary Table 6. Models of sequence evolution for each partition based on corrected Akaike Information Criterion (AICc) of MEGA6[9]

Partition	Model	Gamma	Invariant
COI			
1st codon of *COI*	HKY	n/a	n/a
2nd codon of *COI*	GTR	2.056	n/a
3rd codon of *COI*	TN93	0.894	0.542
28S rRNA	GTR	0.620	0.395
16S rRNA	GTR	0.619	0.426

n/a – not available.

Supplementary References

1. Do, V.T., Tuan, L.Q. & Bogan, A.E. Freshwater mussels (Bivalvia: Unionida) of Vietnam: diversity, distribution, and conservation status. *Freshwater Mollusk Biology and Conservation* **21**, 1–18 (2018).
2. Pfeiffer, J. M., Graf, D. L., Cummings, K. S. & Page, L. M. Molecular phylogeny and taxonomic revision of two enigmatic freshwater mussel genera (Bivalvia: Unionidae incertae sedis: Harmandia and Unionetta) reveals a diverse clade of Southeast Asian Parreysiinae. *Journal of Molluscan Studies*; DOI:10.1093/mollus/eyy028 (2018).
3. Graf, D. L. & Cummings, K. S. The freshwater mussels (Unionoida) of the World (and other less consequential bivalves), updated 9 August 2018. MUSSEL Project Web Site. Available: http://www.mussel-project.net (2018).
4. Lobo, J. *et al.* Enhanced primers for amplification of DNA barcodes from a broad range of marine metazoans. *BMC Ecology* **13,** 34; DOI:10.1186/1472-6785-13-34 (2013).
5. Palumbi, S.R. Nucleic acids II: The polymerase chain reaction. in *Molecular Systematics* (Hillis D.M., Moritz C., Mable B.K., eds.). 205–247 (Sinauer Associates Inc., Sunderland, MA, 1996).
6. Lydeard, C., Mulvey, M., Davis, G. M. Molecular systematics and evolution of reproductive traits of North American freshwater unionacean mussels (Mollusca: Bivalvia) as inferred from 16S rRNA gene sequences. *Philosophical Transactions of the Royal Society B: Biological Sciences* **351,** 1593–1603; DOI:10.1098/rstb.1996.0143 (1996).
7. Jovelin, R. & Justine, J. L. Phylogenetic relationships within the polyopisthocotylean monogeneans (Platyhelminthes) inferred from partial 28S rDNA sequences. *International Journal for Parasitology* **31,** 393–401; DOI:10.1016/S0020-7519(01)00114-X (2001).
8. Talavera, G. & Castresana, J. Improvement of phylogenies after removing divergent and ambiguously aligned blocks from protein sequence alignments. *Systematic Biology* **56**, 564–577; DOI:10.1080/10635150701472164 (2007).
9. Tamura, K., Stecher, G., Peterson, D., Filipski, A. & Kumar, S., MEGA6: Molecular Evolutionary Genetics Analysis version 6.0. *Molecular Biology and Evolution* **30**, 2725–2729; DOI:10.1093/molbev/mst197 (2013).
